# Supplementary material for: Parallel adaptation in autopolyploid Arabidopsis arenosa is dominated by repeated recruitment of shared alleles
Source: Nat Commun. 2021 Aug 17;12:4979. doi: 10.1038/s41467-021-25256-5 (PMC8370997; doi:10.1038/s41467-021-25256-5)
Supplement: Supplementary file 1 — Supplementary information file [file 41467_2021_25256_MOESM1_ESM.pdf]

Parallel adaptation in an autopolyploid *Arabidopsis arenosa* is dominated by repeated recruitment of shared alleles

Konečná *et al.*

Supplementary Method 1. DNA extraction

The samples were washed three times with 2 mL 0.1 M Potassium Phosphate buffer (pH 8.0) by vortexing twice for 10 sec. After the three washes the supernatant was completely removed, and each leaf transferred into a 2 mL tube and stored at -80˚C until further use. Before gDNA extraction we grinded the leaf samples to a powder with ice cold sterile blue pestles (12-141-363, Thermo Fisher Scientific, USA). We extracted gDNA from the powder using the DNeasy PowerPlant Pro® kit (69204, Qiagen, UK) according to the kits manual with one minor alteration: after adding the PD2 solution to the PowerPlant bead tube we processed the samples using a Tissue Lyser II (Qiagen) at 20 Hz for 10 min. We determined the gDNA concentrations using the Qubit 2.0 Fluorometer (Thermo Fisher Scientific, Waltham, USA) dsDNA HS DNA assay kit (Q32851, Thermo Fisher Scientific, Waltham, USA). To assess the size of the extracted gDNA we analysed the fragment sizes using the 2200 TapeStation (Agilent Technologies, Stockport, UK) genomic DNA screen tape (5067-5365, Agilent Technologies, Stockport, UK) and genomic DNA reagents (5067-5366, Agilent Technologies, Stockport, UK).

Supplementary Method 2. Elemental soil concentrations genomic samples

The soil samples were extracted with 8-12 mL of 1M NH4HCO3/ 0.005M DTPA and 2-3 mL of 18.2 MΩcm Milli-Q Direct water (Merck Millipore) (the volume of buffer and water depended on the sample weight) during 1h shaking at 150 rpm on the rotary shaker. Then the samples were gravity filtered through quantitative filter paper (Whatman 42-WHA1442070) and 0.5 mL of filtrate was transferred to Pyrex test tubes and digested in dry block heater with 2 mL trace metal grade nitric acid Primar Plus (Fisher Chemicals) spiked with indium internal standard for 4h at 115°C. Then the samples were diluted with MQ water to 20 mL. Elemental analysis was performed with an ICP-MS, PerkinElmer NexION 2000 equipped with Elemental Scientific Inc. autosampler, in the collision mode (He). Liquid reference material composed of pooled samples was prepared before the beginning of sample run and was used throughout the whole samples run. It was run after every ninth sample to correct for variation within ICP-MS analysis run[1]. The calibration standards (with indium internal standard and blanks) were prepared from single element standards (Inorganic Ventures; Essex Scientific Laboratory Supplies Ltd, Essex, UK) solutions. Sample concentrations were calculated using external calibration method within the instrument software. Further data processing was performed using Microsoft Excel spreadsheet.

Supplementary Method 3. Elemental leaf concentrations samples from reciprocal transplant experiment

Due to very small amounts of samples in units to tens of mg, the samples were decomposed prior to the analysis using the microwave oven Speedwave ®Xpert (Berghof, Germany, maximal applied power 2000 W) with a multi_tube system. The plant tissue (2 replicates, 8 – 50 mg according to the available sample amount) was inserted into digestion tubes and treated with 2 mL of subboilingly destilled (Berghof, Germany) nitric acid (per analysis, Lachner, the Czech Republic) under the following conditions: 10 min hold on 170°C, 30 % of maximal power, 10 min on 200°C, 30 % of power, 30 min on 30°C, 0 % of power. The

mineralised samples were filled up to the final volume of 10 mL with deionised water (conductivity 0.055 µS/cm, Evoqua Water Technologies, Germany).

The elemental analysis of Ca, Mg, Co, Cr and Ni from desiccated leaf tissue was carried out using the sequential, radially viewed ICP OES spectrometer INTEGRA 6000 (GBC, Dandenong Australia) equipped with the ultrasonic nebulizer U5000AT+ (Teledyne Cetac Technologies, the USA), concentric nebulizer (2 mL/min) and a glass cyclonic spray chamber (both Glass Expansion, Australia). The samples were decomposed prior to the analysis. The analytical lines used were: Mg 285.2213 nm, Ca 422.673 nm, Ni 221.647 nm, Co 238.892 nm and Cr 267.716 nm. The operation conditions of the ICP OES analysis were as follows: sample flow rate 1.5 mL/min, plasma power 1000 W, plasma, auxiliary and nebulizer gas flow rates 10, 0.4, and 0.52 L/min, respectively, photomultiplier voltage 600 V for Ni, Co and Cr and 350 V for Ca and Mg, view height 6.5mm, three replicated reading on_peak 1 s, fixed point background correction. The multielemental standards containing 10 – 5 – 1 – 0.5 – 0.1 mg/L of Mg and Ca and 0.1 – 0.05 – 0.01 – 0.005 – 0.001 mg/L Ni, Co and Cr were used for instrument calibration. The external calibration standards were prepared using standard solutions of Mg, Ca, Ni, Co and Cr all containing 1 g/L (SCP, Canada). The limits of detection (concentration equal to three times the standard deviation at the point of the background correction) were 0.1 µg/L for Ni, Co and Cr and 2 µg/L for Mg and Ca. Certified reference material (Bush twigs and leaves GBW 07602 from the China National Analysis Center for Iron and Steel, Beijing) was used to validate the method and for the quality control.

Supplementary Table 1. Details on the sampled populations.

Pop code	Pop	Ploidy	Pop name	N ind ionomics /genome	Bedrock	Altitude	Lat	Lon	Country
BOR	S1	4x	Borovsko	8/8	serpentine	416	49.68381	15.133255	CZ
STG	S2	4x	Steinegg	8/7	serpentine	414	48.62993	15.54256	AT
GUL	S3	4x	Gulsen	8/8	serpentine	628	47.28167	14.92764	AT
OPP	S4	4x	Oppenberg	8/8	serpentine	1750	47.46403	14.23989	AT
PER	S5	4x	Pernegg	8/8	serpentine	540	47.35512	15.33697	AT
VLA	N1	4x	Vlastejovice	8/8	siliceous	345	49.73496	15.17484	CZ
FUG	N2	4x	Fuglau	8/8	siliceous	436	48.63149	15.55723	AT
ING	N3	4x	Ingeringgraben	8/8	siliceous	950	47.28405	14.68154	AT
VOR	N4	4x	Vorberg	8/8	siliceous	1010	47.49876	14.16964	AT
HOC	N5	4x	Hochlantsch	8/7	siliceous	545	47.37	15.38667	AT

Abbreviations: Pop – population; N ind ionomics/genome – number of individuals used for ionomics/genomic analyses; Lat – latitude; Lon – longitude.

Supplementary Table 2. Summary of datasets and filtrations for genomic analyses.

Analysis	Dataset	Input filtrations
**Faststructure**	4dg SNPs	MFFG 0.2, DP < 8, pruning over 1kbp windows and 10kbp distance between the windows, MAF < 0.05
**PCA**	4dg SNPs	MFFG 0.2 and DP < 8
**Treemix**	4dg SNPs	MFFG 0.2 and DP < 8
**Pairwise F_{ST}, nucleotide diversity, Tajima's D, Fastsimcoal**	4dg, SNPs + invariants	MFFG 0.2 and DP < 8 at the population level
**F_{ST} scans**	all SNPs	MFFG 0.2 and DP < 8 at the population level
**LFMM**	all SNPs	missing data filtered out, MAF < 0.05
**Differentiation of regions containing TE variants**	all SNPs	MAF < 0.05, MFFG 0.2 and DP < 8
	and TE variants	
**DMC**	all SNPs annotated	MFFG 0.2 and DP < 8
**DMC (neutral data)**	4dg SNPs	MFFG 0.2 and DP < 8

Number of sites before filtrations in brackets:
All SNPs (11,744,200): biallelic SNPs pre-filtered following GATK best parctices
4dg sites (4,720,044): fourfold-degenerate sites
All SNPs annotated (8,808,984): same as all sites with available annotation for SNPs retained from SnpEff.
MAF: minor allele frequency
MFFG: maximum fraction of filtered genotypes (reflecting missingness)
DP: read depth

Supplementary Table 3. Summary of SNP and TE variation for each population pair.

pop	n SNPs[1]	pop. pair	n SNPs[1]	n SNPs[2]	n TEs[3]	n of 1kbp outlier windows (SNPs)	n of 1kbp outlier windows (TEs)
S1	395,760	S1-N1	547,071	4,320,217	9,798	953	91
N1	438,869						
S2	449,103	S2-N2	561,047	4,458,385	10,197	954	94
N2	432,398						
S3	406,035	S3-N3	505,296	3,410,315	10,183	957	91
N3	365,023						
S4	384,194	S4-N4	487,623	3,842,682	10,112	935	93
N4	389,774						
S5	418,341	S5-N5	452,303	3,028,157	-	726	-
N5	354,981						

[1] 4dg SNPs MFFG 0.2 and DP < 8
[2] all SNPs MFFG 0.2 and DP < 8
[3] filtered TE variants

Supplementary Table 4. Summary of population means of selected environmental variables (Temp = annual mean temperature, Prec = annual precipitation (both from average for 1970-2000 and spatial resolution ~1km^2), Elev = elevation. For soil variables, min, **mean**, and max ppm concentrations are presented in separate lines for each population).

Pop	Temp [°C]	Prec [mm]	Elev [m]	Mg [ppm]	Ni [ppm]	Co [ppm]	Ca/Mg	Ca [ppm]	P [ppm]	S [ppm]	K [ppm]
S1	7.8	605.0	416.0	1938.2	62.4	2.9	0.3	825.4	30.3	34.6	97.6
				**2324.5**	**136.6**	**5.7**	**0.6**	**1447.5**	**81.7**	**82.8**	**213.0**
				3338.2	248.8	9.8	1.2	2473.4	168.8	149.2	354.1
S2	8.2	694.0	414.0	2523.6	101.4	6.1	0.3	1036.5	39.2	48.1	207.6
				**3877.7**	**156.3**	**9.3**	**0.5**	**1730.9**	**67.2**	**116.9**	**294.6**
				4983.2	259.9	17.5	0.6	2208.3	128.8	251.9	398.9
S3	6.8	983.0	628.0	662.9	18.9	2.3	0.5	332.1	73.7	24.0	52.8
				**2247.2**	**50.3**	**6.8**	**1.1**	**2437.9**	**213.4**	**88.7**	**614.3**
				4836.1	117.1	15.2	2.5	4801.6	358.0	226.1	1103.1
S4	1.4	1503.0	1750.0	971.1	37.1	2.7	0.3	956.7	22.7	49.0	67.2
				**3289.5**	**99.3**	**7.5**	**0.7**	**1890.1**	**87.5**	**145.6**	**209.5**
				4989.1	141.2	13.6	1.3	4035.5	150.9	427.5	357.2
S5	7.2	896.0	540.0	1115.4	19.3	1.9	0.5	920.3	19.4	42.1	118.5
				**2013.7**	**70.0**	**4.0**	**1.3**	**2498.5**	**62.7**	**99.3**	**248.9**
				3326.5	132.5	6.9	2.5	5492.2	138.2	389.6	452.7
N1	8.2	581.0	345.0	143.6	4.7	0.2	2.9	601.4	22.5	44.7	62.7
				**246.1**	**7.1**	**0.4**	**3.5**	**846.2**	**48.2**	**64.4**	**165.9**
				347.7	12.2	0.7	4.2	1154.5	70.5	94.5	353.1
N2	8.3	684.0	436.0	167.5	0.7	1.0	2.7	665.4	25.6	11.3	100.6
				**223.7**	**1.1**	**1.9**	**4.4**	**923.0**	**54.0**	**40.3**	**176.2**
				289.8	1.8	3.5	8.5	1425.3	71.8	78.6	291.1
N3	4.3	1200.0	950.0	176.8	4.2	1.3	1.2	1043.1	68.0	67.2	145.2
				**771.8**	**21.1**	**3.6**	**3.3**	**2039.5**	**137.8**	**100.7**	**574.3**
				1443.2	40.4	7.5	6.1	5137.7	230.8	160.2	1237.6
N4	5.3	1299.0	1010.0	29.3	0.2	0.2	12.7	704.6	8.7	8.4	25.8
				**41.8**	**0.5**	**0.5**	**21.8**	**835.4**	**22.4**	**28.7**	**94.8**
				86.5	0.8	0.7	26.4	1102.9	55.5	53.8	221.2
N5	6.8	914.0	545.0	773.4	1.2	0.9	1.7	2889.9	37.8	70.8	310.7
				**2311.9**	**1.8**	**2.1**	**2.6**	**5335.2**	**175.0**	**156.4**	**792.9**
				3662.3	2.9	4.1	4.2	7508.3	355.2	428.7	1511.5

Supplementary Table 5. Gene coding loci exhibiting excessive differentiation (1% outlier F_{ST}) between particular S-N population pair in *A. arenosa* which have been also identified as canddiates for serpentine adaptation in other available plant studies.

Species	Family	***A. arenosa* S1-N1**	***A. arenosa* S2-N2**	***A. arenosa* S3-N3**	***A. arenosa* S4-N4**	***A. arenosa* S5-N5**
Arabidopsis lyrata[2]	Brassicaceae	AT3G15730, AT4G19440, AT5G09650, AT1G31120, AT1G69730, AT5G03570, AT4G19960	AT1G72560, AT4G03560, AT4G19960	AT2G46140, AT3G01310, AT4G32640, AT4G12430, AT1G51310, AT1G31120, AT4G03560, AT4G19960	AT2G24070, AT3G46520, AT1G51310, AT4G03560, AT5G03570	AT4G34450, AT1G69730, AT5G03570
Alyssum serpyllifolium[3]	Brassicaceae	AT5G03570			AT5G03570	AT5G03570
Mimulus guttatus[4]	Phrymaceae				AT4G19880, AT4G19670	

[1](Turner *et al.*[2]), N of candidates = 62

[2](Sobczyk *et al.*[3]) N of candidates = 2

[3](Selby[4]), N of candidates = 10

Supplementary Table 6. Parameters used in parallel selection modelling in DMC.

**Parameter**	**Parallel selection scenario**	**Values used**
selection coefficient	all scenarios	0.0001, 0.001, 0.01, 0.05, 0.1, 0.2, 0.5
migration rates	migration	1e-07, 1e-06, 0.00001, 0.0001, 0.001, 0.01
initial allele frequencies prior to selection	standing variation	2.5e-06, 0.00001, 0.0001, 0.001, 0.01
times for which allele was standing prior to onset of selection[1]	standing variation	100, 1000, 5000, 10000, 30000, 50000, 100000

[1] This time range spans from the low CI of the youngest split between S and N populations inferred by our coalescent simulations to approximately three times of the origin of the autotetraploid cytotype as inferred for *A. arenosa* by previous range-wide studies[5,6].

We used effective population size $N_e = 100{,}000$ calculated from the mean nucleotide diversity of our populations 0.029 following the equation $N_e = \pi/8\mu$ ($\mu = 4.3e^{-8}$ from Arnold et al.[7]).

We used recombination rate of $3.7e^{-8}$ estimated for the reference species *A. lyrata* previously[8].

Supplementary Table 7. Summary of allele frequencies of non-synonymous substitutions in the *TPC1* locus in all *A. arenosa* populations resequenced in this study (separately for each S pop., allele number (AN) = 28-32, and summed across all N pops, AN = 156). The high-frequency serpentine-specific alleles are highlighted in bold.

Position	Substitution	S1	S2	S3	S4	S5	N
scaffold_6:23047864	Phe79Leu	0.000	0.594	0.781	0.719	0.844	0.526
scaffold_6:23047750	Gln85Lys	0.000	0.000	0.000	0.000	0.781	0.295
scaffold_6:23047666	Asn113Asp	0.000	0.000	0.000	0.000	0.594	0.205
scaffold_6:23047662	Val114Ala	0.000	0.000	0.000	0.000	0.656	0.314
scaffold_6:23047015	Leu190Ile	0.906	0.875	1.000	0.938	1.000	0.929
scaffold_6:23046782	His200Asp	0.563	0.688	0.969	0.688	1.000	0.750
scaffold_6:23046751	Gly210Val	0.563	0.688	0.969	0.719	1.000	0.737
scaffold_6:23046295	Ile300Val	0.656	0.813	1.000	1.000	1.000	0.853
scaffold_6:23046128	Arg320Lys	0.594	0.750	0.969	0.781	1.000	0.769
scaffold_6:23045888	Glu349Gln	0.594	0.688	0.969	0.781	1.000	0.744
scaffold_6:23045881	Asn352Thr	0.688	0.781	1.000	1.000	1.000	0.840
scaffold_6:23045354	Lys409Gln	0.594	0.688	0.969	0.688	1.000	0.756
scaffold_6:23045270	Val437Ile	0.656	0.844	1.000	1.000	1.000	0.846
scaffold_6:23044774	Ala492Ser	0.594	0.000	0.906	0.000	0.000	0.622
scaffold_6:23044106	Ile582Val	0.719	0.813	1.000	1.000	0.906	0.853
scaffold_6:23044055	Leu599Met	0.594	0.563	0.969	0.750	0.781	0.692
scaffold_6:23043812	Val630Ile	0.000	0.000	0.031	0.000	0.031	0.090
**scaffold_6:23043812**	**Val630Leu**	**0.000**	**0.000**	**0.938**	**0.000**	**0.469**	**0.000**
**scaffold_6:23043812**	**Val630Tyr**	**0.000**	**0.000**	**0.000**	**0.659**	**0.000**	**0.000**
scaffold_6:23043385	Asn682Lys	0.000	0.719	0.969	0.750	0.938	0.756
scaffold_6:23043108	Thr700Ser	0.000	0.000	0.719	0.000	0.656	0.487

Supplementary Table 8. Chi-squared test for rotamer positioning of 630Tyr *TPC1* alllele in a homodimer or heterodimer. Position is referred to as either 'in' when the residue points towards the channel or 'out' when the residue points away from the channel.

**Homodimer**	**Expected value**	**Actual value**
1 in, 1 out	33.3	35
2 in	33.3	20
2 out	33.3	55
p-value		0.000058
**Heterodimer**		
In	50	41
Out	50	59
p-value		0.0718606
**Homo**		
1 in, 1 out	45	35
2 out	45	55
p-value		0.035015

Supplementary Table 9. Pairwise matrix of Hudson's F_{ST} among all populations inferred from genome-wide set of 4dg SNPs.

	S1	N1	S2	N2	S3	N3	S4	N4	S5
S1									
N1	0.069								
S2	0.0752	0.0673							
N2	0.0855	0.0772	0.0291						
S3	0.1093	0.0983	0.0819	0.0917					
N3	0.1266	0.1169	0.0985	0.1081	0.0849				
S4	0.1161	0.1072	0.0882	0.0982	0.0772	0.0744			
N4	0.1133	0.1039	0.0859	0.0954	0.0758	0.0723	0.0566		
S5	0.1043	0.0929	0.0767	0.0864	0.0654	0.0938	0.0851	0.0823	
N5	0.0989	0.0882	0.0717	0.0818	0.0641	0.0923	0.0845	0.0812	0.0467

Supplementary Table 10. Primers used for amplifying the focal part of the TPC1 locus (scaffold_6:23,043,556–23,043,854 bp in the reference).

Primer	Sequence (5'->3')
forward primer	CAAATTCAACAGAAGTAAAATAGTGATGACG
reverse primer	CTATCTATTGTTCAACTTCAATGACTACCCC

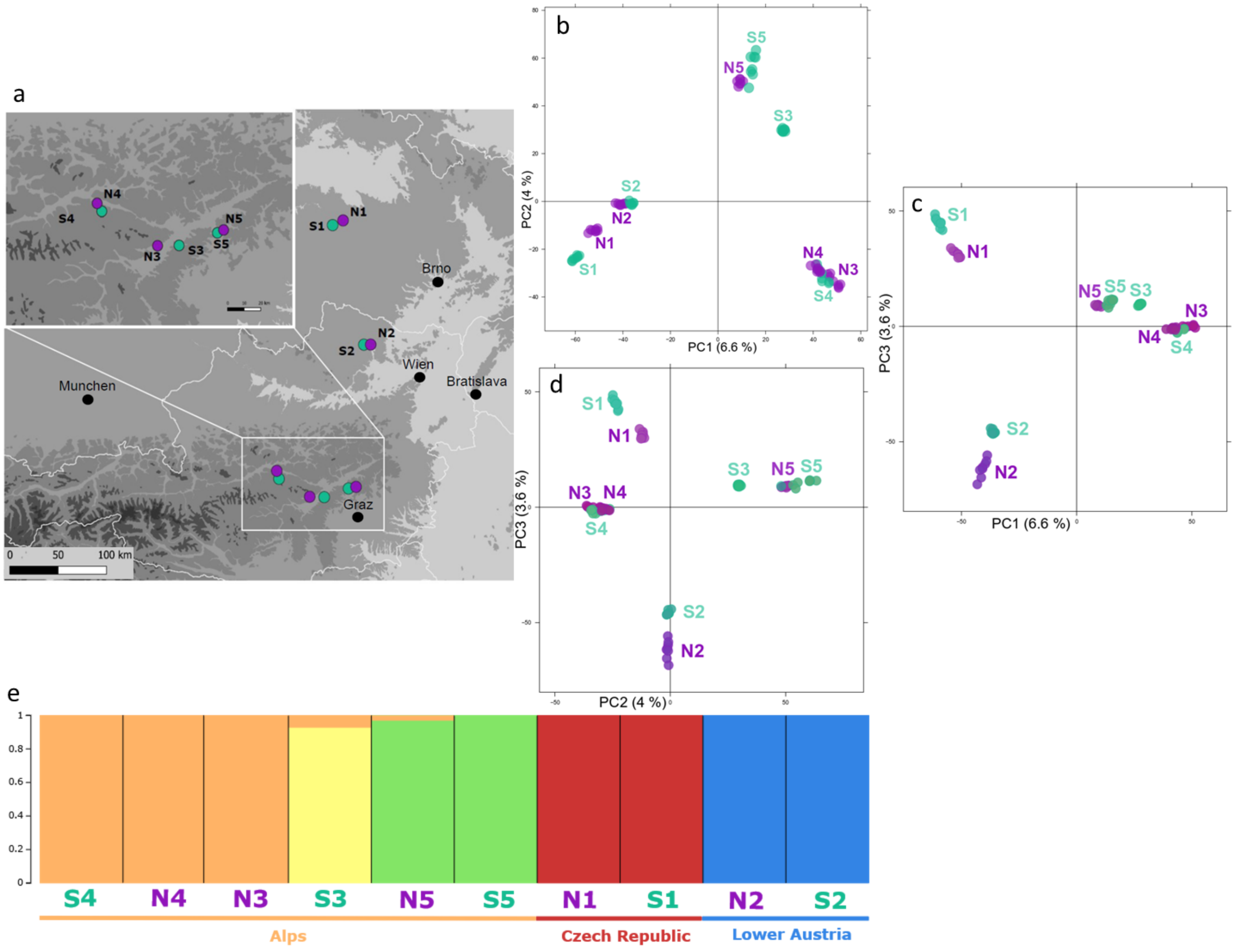

Supplementary Fig. 1. Geographic distribution and genetic structure of sampled autotetraploid *A. arenosa* populations in Central Europe inferred by analysis of 4dg SNPs. a) Locations of the populations (Map drawn by V. Konečná). b-d) Principal component analyses based on ~1M fourfold-degenerate SNPs showing relationships among all individuals. e) Proportional assignment of individuals to five clusters (corresponding to the number of population pairs) inferred by fastStructure (9,923 LD-thinned 4dg SNPs). S – serpentine, N – non-serpentine.

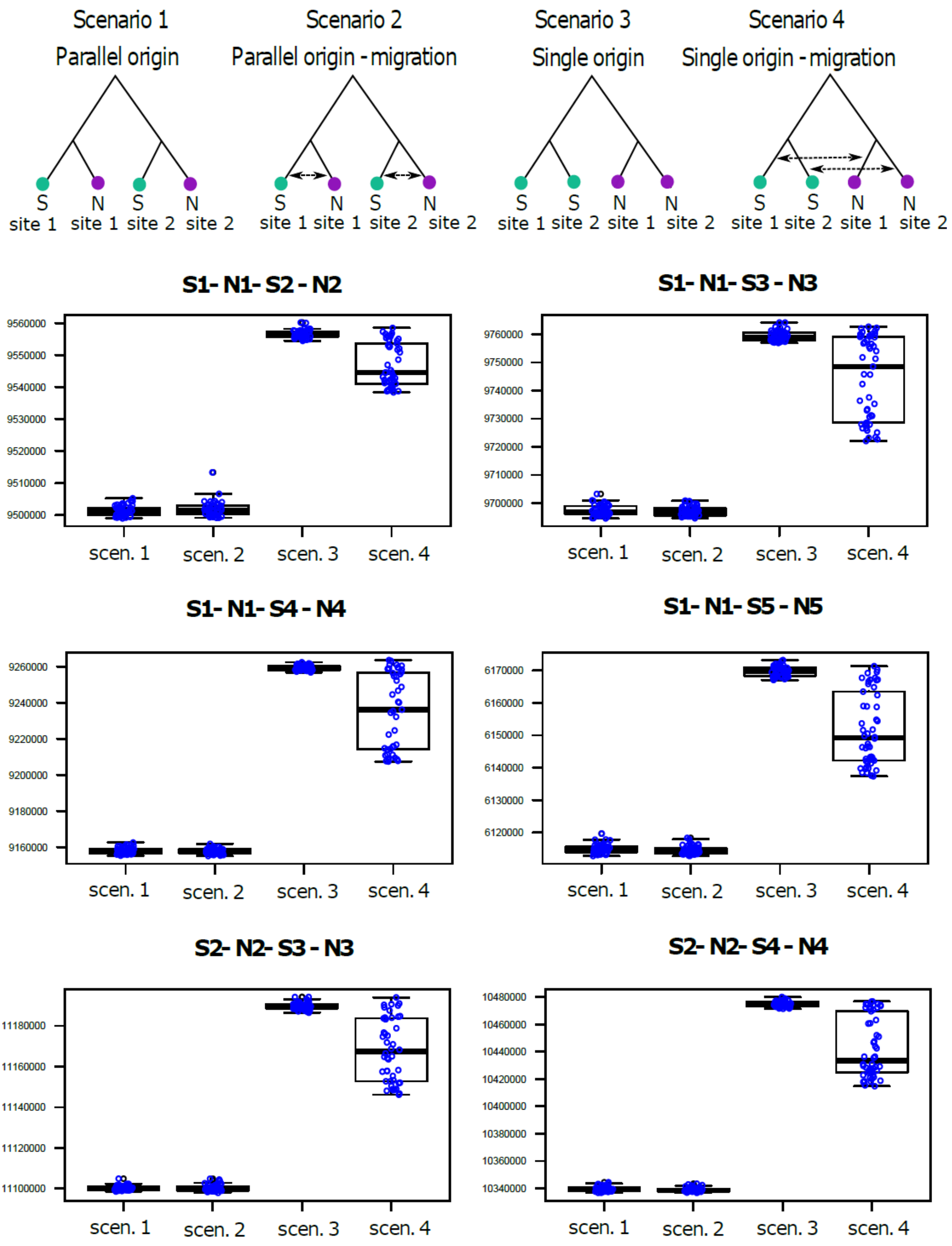
Scenario 1
Parallel origin
Scenario 2
Parallel origin - migration
Scenario 3
Single origin
Scenario 4
Single origin - migration
S site 1
N site 1
S site 2
N site 2
S1- N1- S2 - N2
S1- N1- S3 - N3
S1- N1- S4 - N4
S1- N1- S5 - N5
S2- N2- S3 - N3
S2- N2- S4 - N4
scen. 1
scen. 2
scen. 3
scen. 4

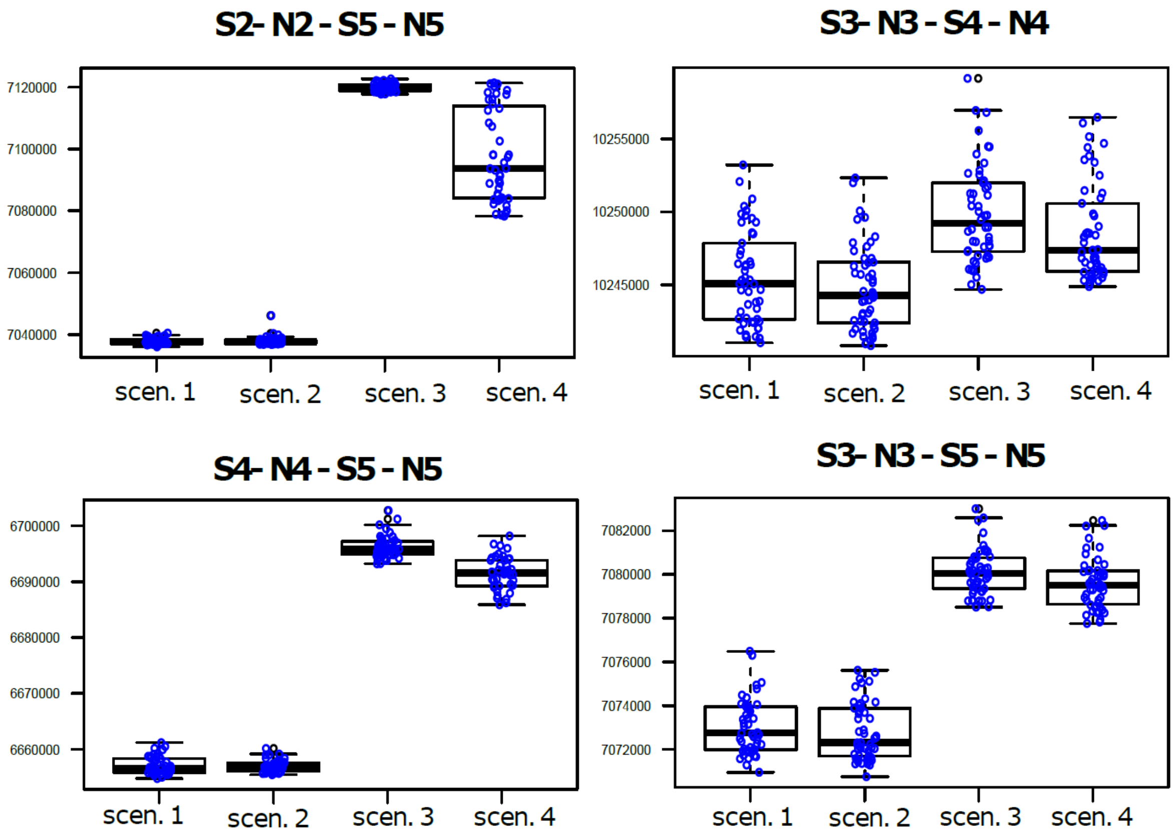

Supplementary Fig. 2. Comparison of Akaike information criteria (AIC) across four scenarios approximating the origin of serpentine populations in pairs of geographically proximal serpentine and non-serpentine populations. We iterated all pairs of serpentine (S) and non-serpentine (N) populations in a pairwise manner (i.e. 10 combinations). Each scenario was simulated by independent 50 fastsimcoal runs, the corresponding distribution of the AIC values over these 50 runs (blue dots) is summarized by the boxplots. The topologies of the evaluated scenarios are depicted on top. Consistently over all possible pairwise iterations the scenario of parallel colonisation of serpentine at each site was more likely than single origin of each edaphic type. Note that subsequent gene flow between substrate types within each S-N population pair was unlikely as the assumption of migration within each population pair had not significantly improved the model fit (scenario 1 vs. scenario 2 comparison). Central line denotes median; error bars depict standard deviation.

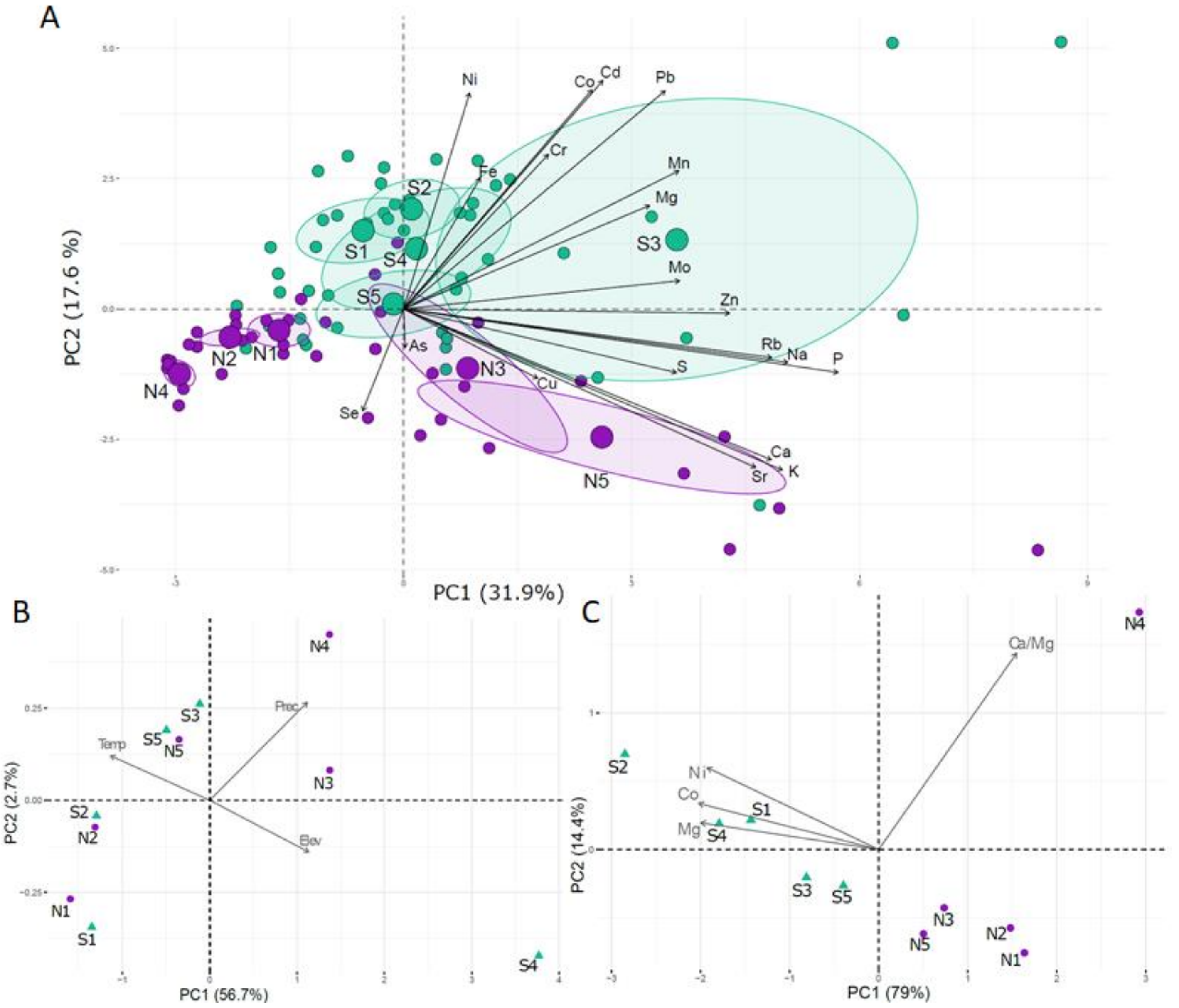

Supplementary Fig. 3. Environmental differentiation of the sampled serpentine (S, green dots) and non-serpentine (N, violet dots) sites displayed by unconstrained ordination (principal component analysis, PCA). a) Overall soil differentiation shown by PCA based on standardized concentrations of 20 elements recorded in rhizosphere soil of each sampled individual (95% confidence ellipses around population means) b) Lack of climatic differentiation between sister S and N populations shown by PCA based on three environmental variables (Temp = annual mean temperature, Prec = annual precipitation (both from average for 1970-2000 and spatial resolution ~1km^2), Elev = elevation. c) PCA based on the four soil variables significantly differentiating S and N soils (Ca/Mg, Co, Mg, and Ni) showing population means.

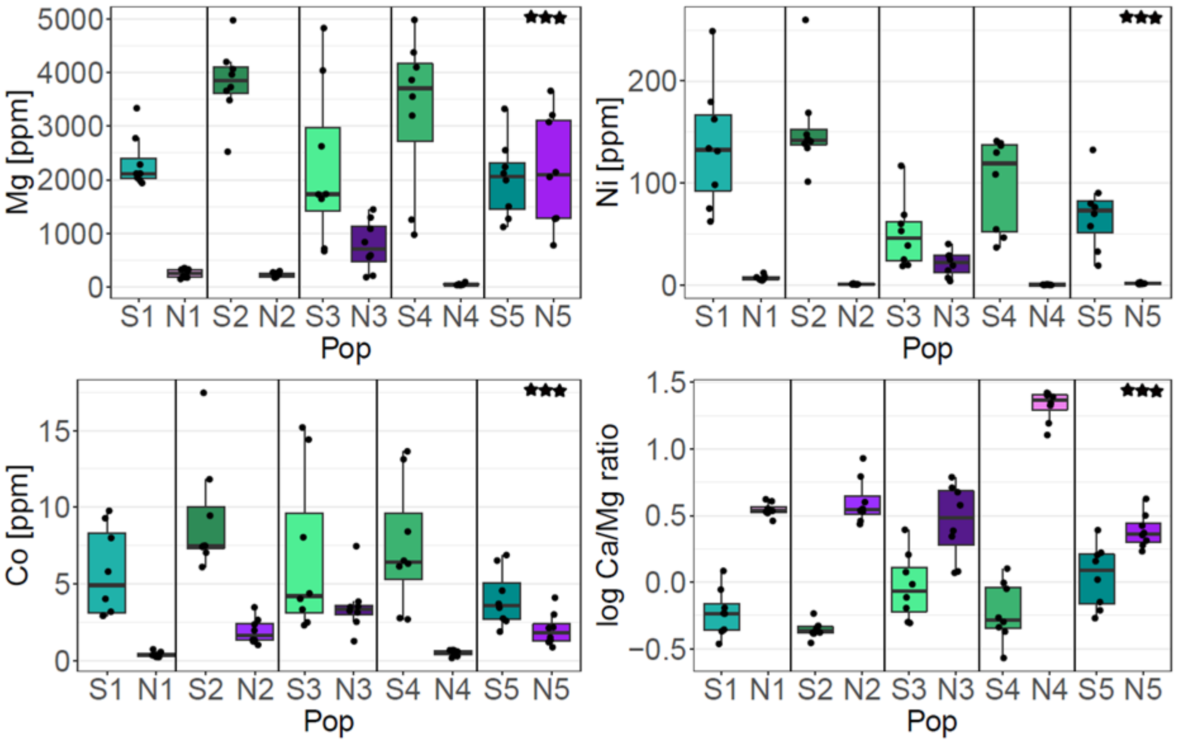

Supplementary Fig. 4. Variation in the concentrations of soil Mg, Ni, Co and Ca/Mg ratio (soil associated with each of the 80 sampled individuals). Note: Outlier S4_5 sample for Co concentration of 28.75 ppm was excluded to aid visibility. Differences between S and N soils in concentration of each element were tested by one-way ANOVA taking population pair as a random variable. Mg: $F_{1,77} = 71.7$, $p = 1.29e\text{-}12$, Ni: $F_{1,77} = 117.4$, $p = 2e\text{-}16$, Co: $F_{1,77} = 54.3$, $p = 1.65e\text{-}10$, and Ca/Mg ratio: $F_{1,77} = 26.5$, $p = 1.94e\text{-}06$. Central line denotes median; error bars depict standard deviation.

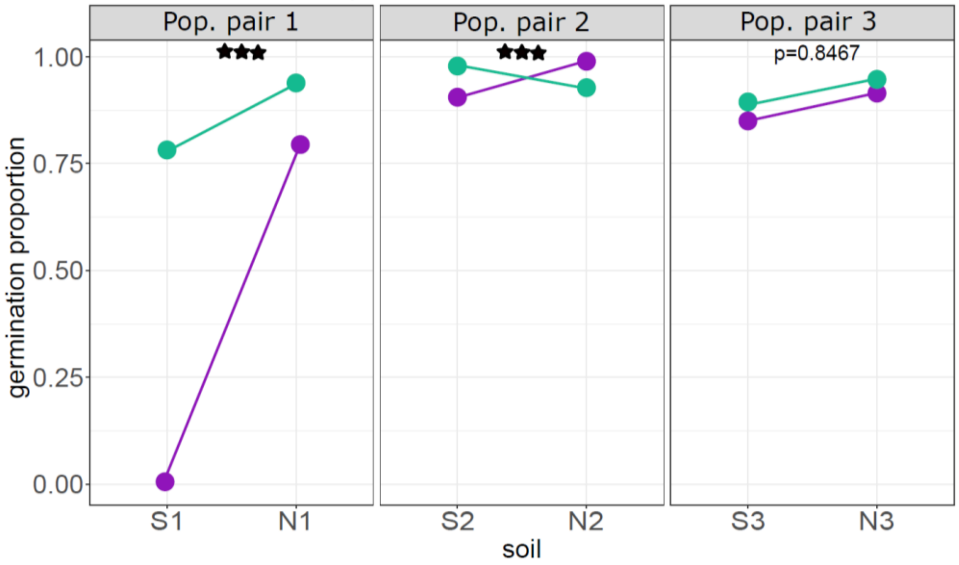

Supplementary Fig. 5. Differences in germination proportion in the originally serpentine (S, green) and non-serpentine (N, violet) populations transplanted to their native and alternative soil. Dots represent mean values across ~ 11 replicates (seed families) per treatment*population. Significances of the soil_treatment * soil_origin interaction terms were estimated by GLM with binomial errors, S1-N1 pair (χ^2= 57.764, p = 2.955e-14), S2-N2 pair (χ^2= 18.4083, p = 1.783e-05), and S3-N3 pair (χ^2= 0.0374, p = 0.8467).

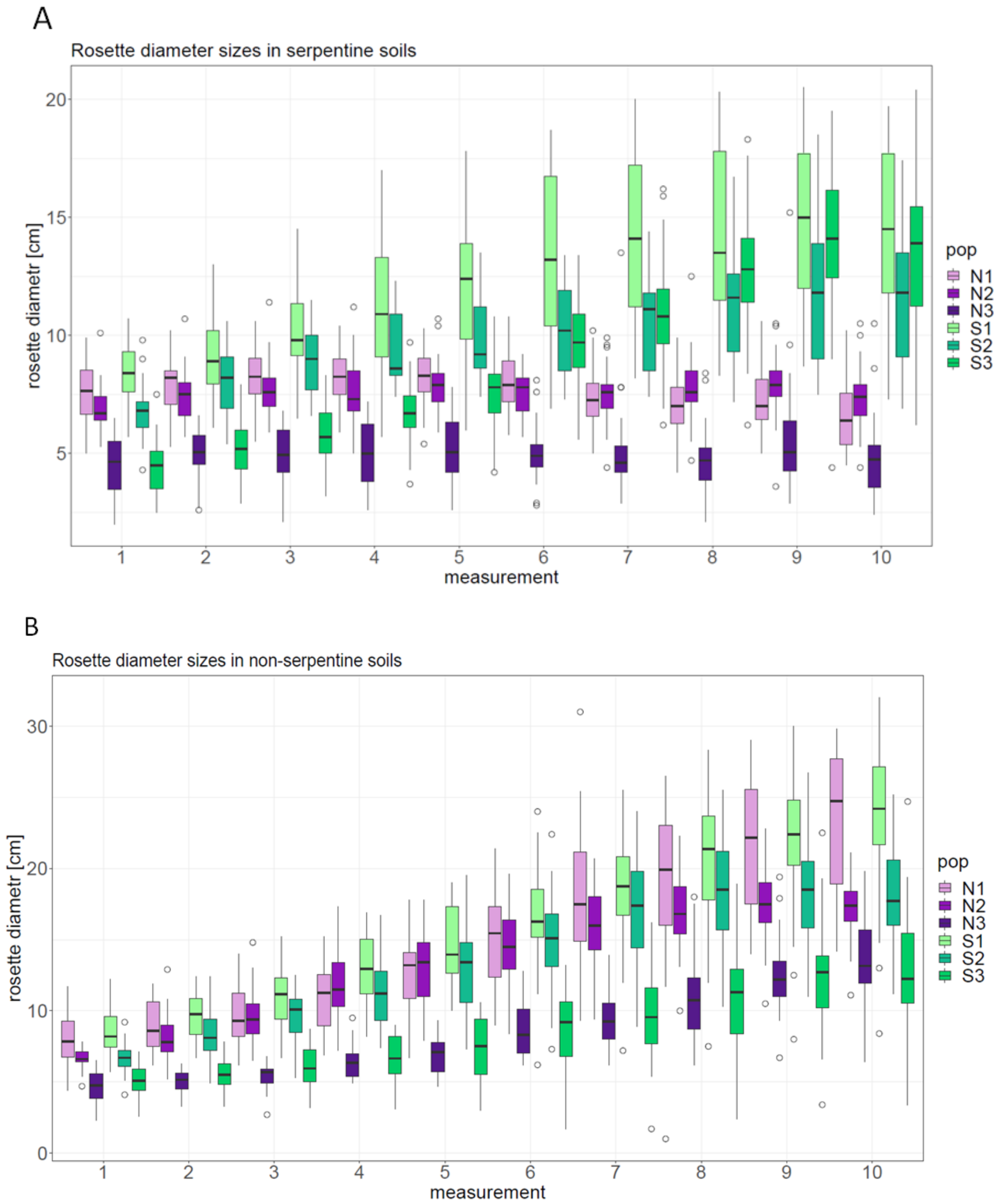

Supplementary Fig. 6. Differences in rosette diameter sizes of the individuals from the originally serpentine (S, green) and non-serpentine (N, violet) populations cultivated in serpentine (a) and non-serpentine soils (b). Data from three population pairs showing entire five-week period of cultivation. Note: rosettes were measured twice a week until all populations reached a plateau in the growth; ~ 25 replicates per population and treatment were cultivated. There was no mortality during the cultivation. Central line denotes median; error bars depict standard deviation.

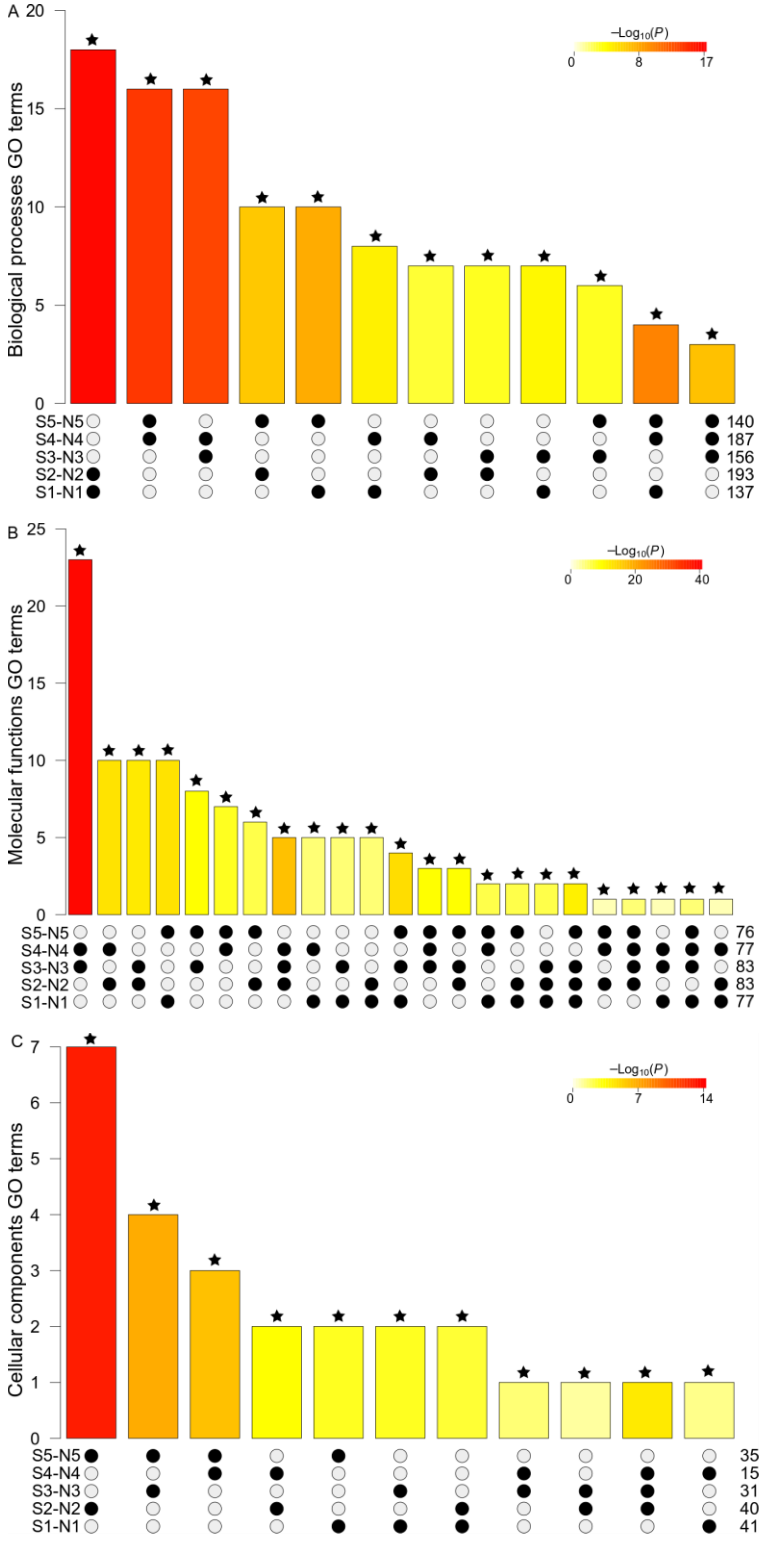

Supplementary Fig. 7. Functional parallelism inferred from significant intersection among lists of enriched gene ontology terms (identified from divergence outlier loci within each population pair), across all population pairs (1- 5); (a) biological processes, (b) molecular functions, (c) cellular components; significant (p < 0.05) overlaps are marked with asterisks; the results of one-sided Fisher's exact test are available in Supplementary Data 4.

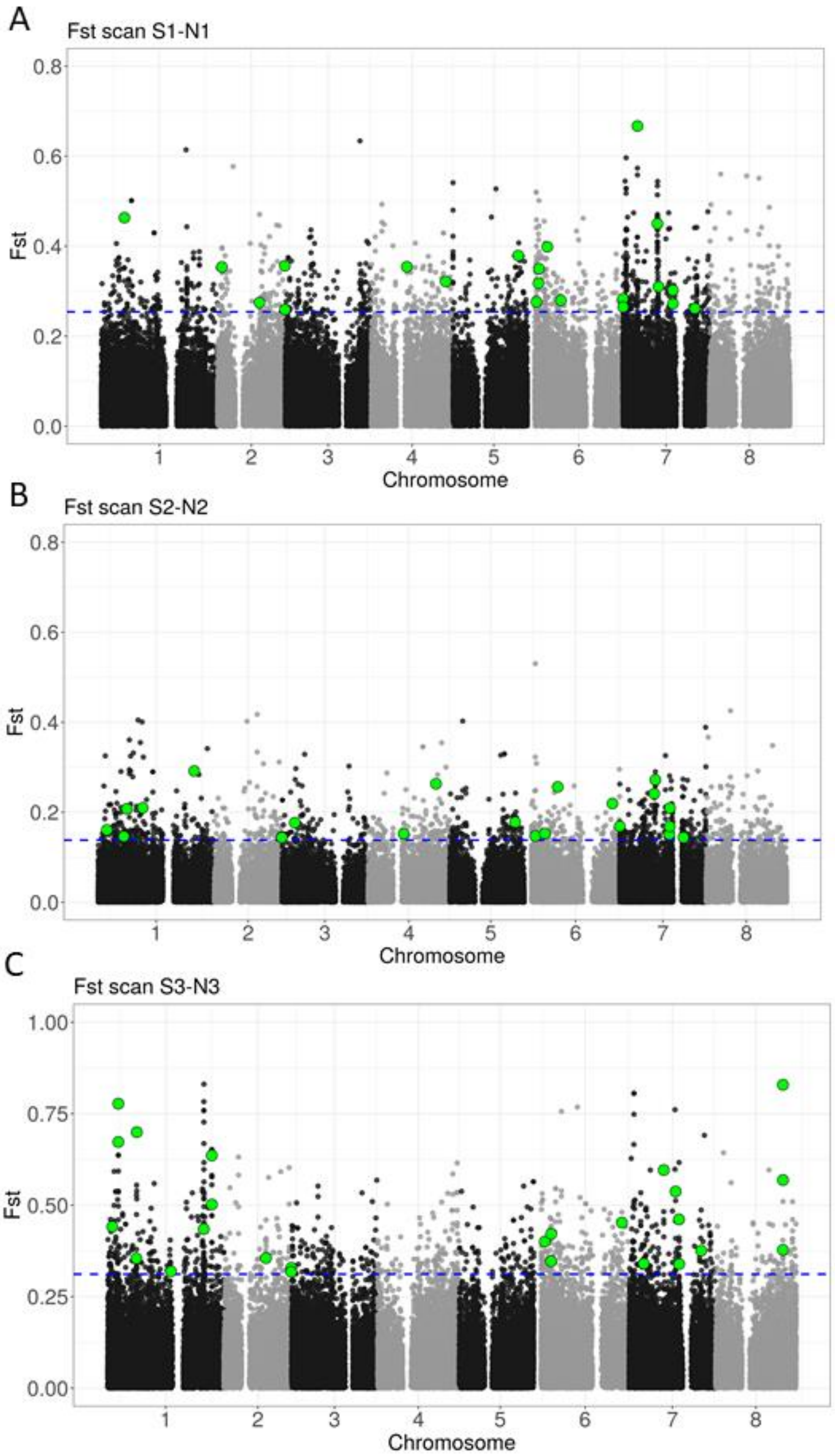
A
Fst scan S1-N1
Fst
0.0
0.2
0.4
0.6
0.8
1
2
3
4
5
6
7
8
Chromosome
B
Fst scan S2-N2
Fst
0.0
0.2
0.4
0.6
0.8
1
2
3
4
5
6
7
8
Chromosome
C
Fst scan S3-N3
Fst
0.00
0.25
0.50
0.75
1.00
1
2
3
4
5
6
7
8
Chromosome

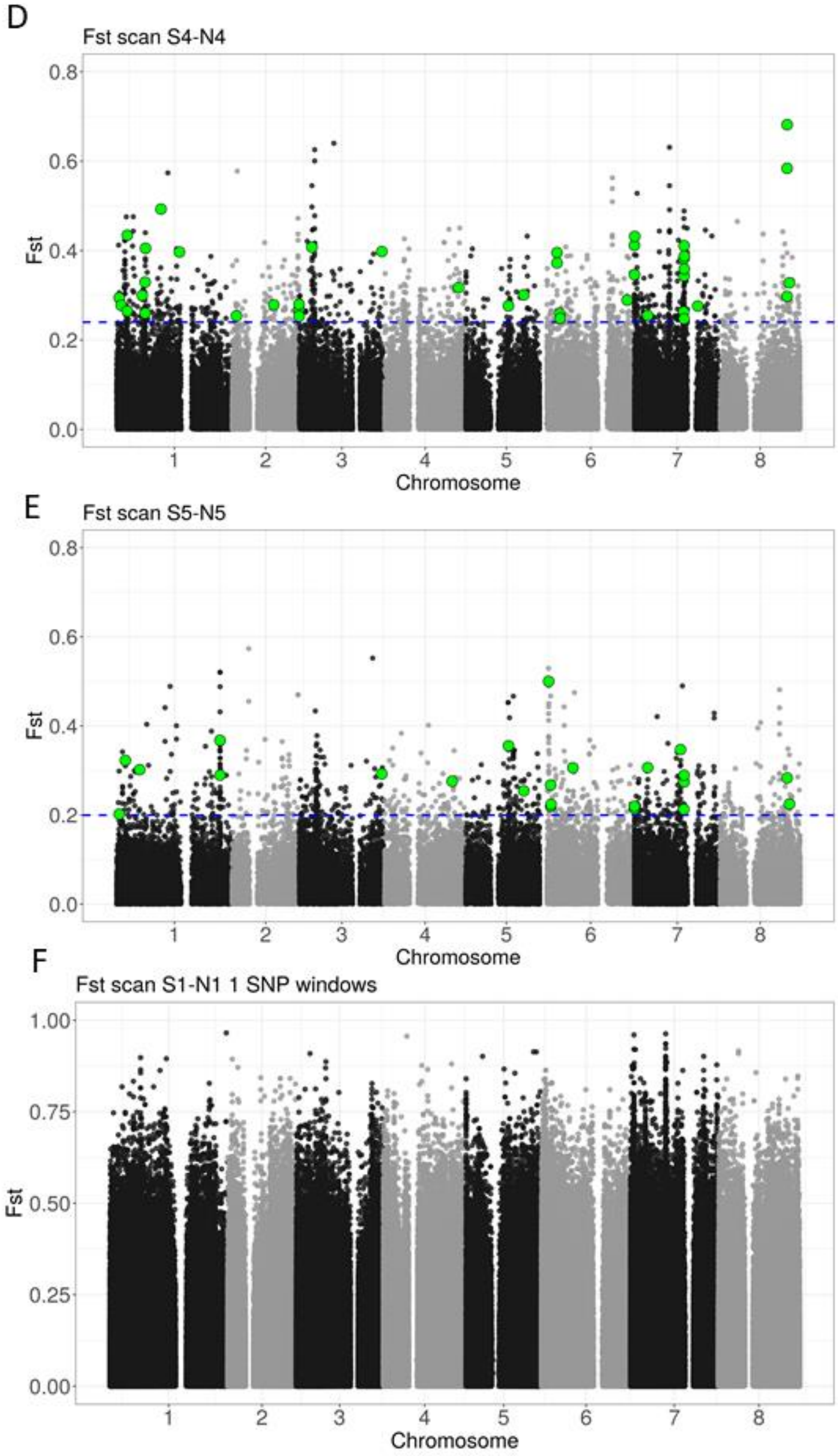
D
Fst scan S4-N4
0.8
0.6
0.4
0.2
0.0
Fst
1
2
3
4
5
6
7
8
Chromosome
E
Fst scan S5-N5
0.8
0.6
0.4
0.2
0.0
Fst
1
2
3
4
5
6
7
8
Chromosome
F
Fst scan S1-N1 1 SNP windows
1.00
0.75
0.50
0.25
0.00
Fst
1
2
3
4
5
6
7
8
Chromosome

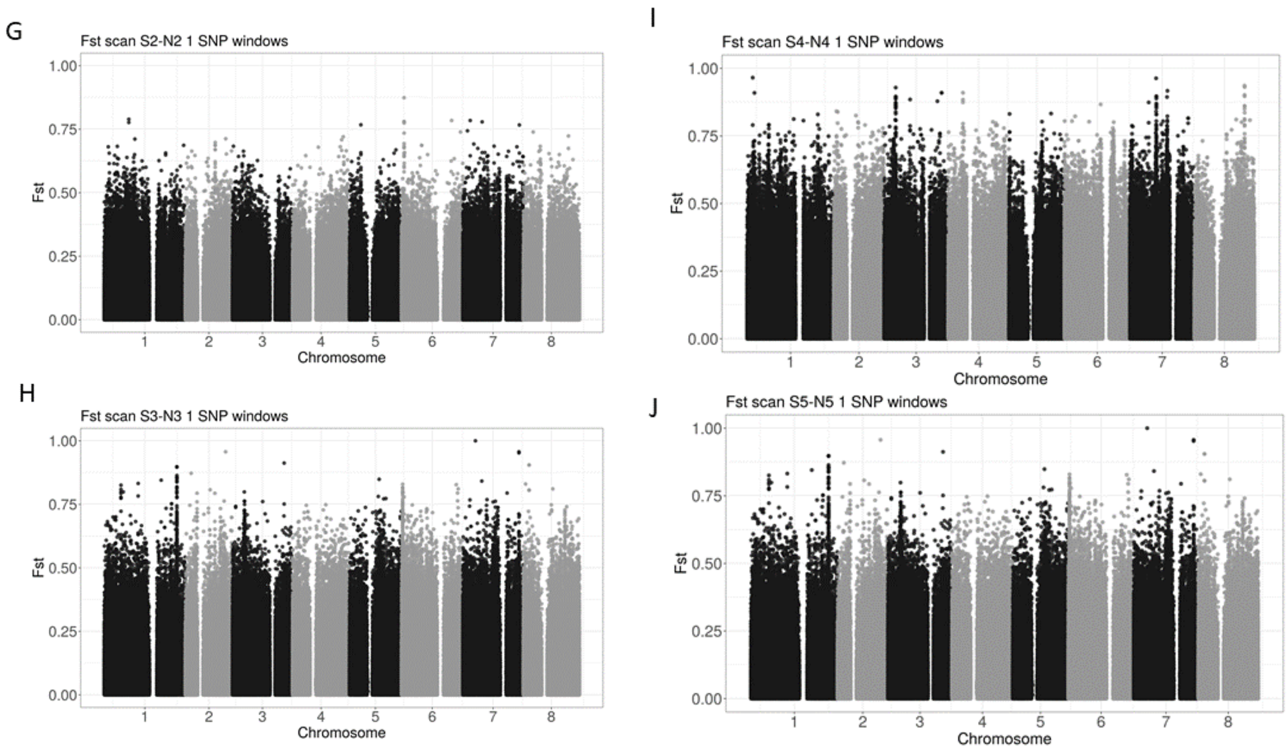

Supplementary Fig. 8. Window-based genomic differentiation between serpentine (S) and non-serpentine (N) population in each population pair (1–5, a–e, respectively) quantified by F_{ST}[9] calculated within 1 kbp windows (a-e); and per one SNPs windows (f-j) spanning the genome. Blue dashed line shows the upper 1% quantile, and green dots highlight position of the window with the highest differentiation value for each of the 61 serpentine adaptation candidate genes to highlight the position of all these loci along the genome (inferred downstream, see the main text).

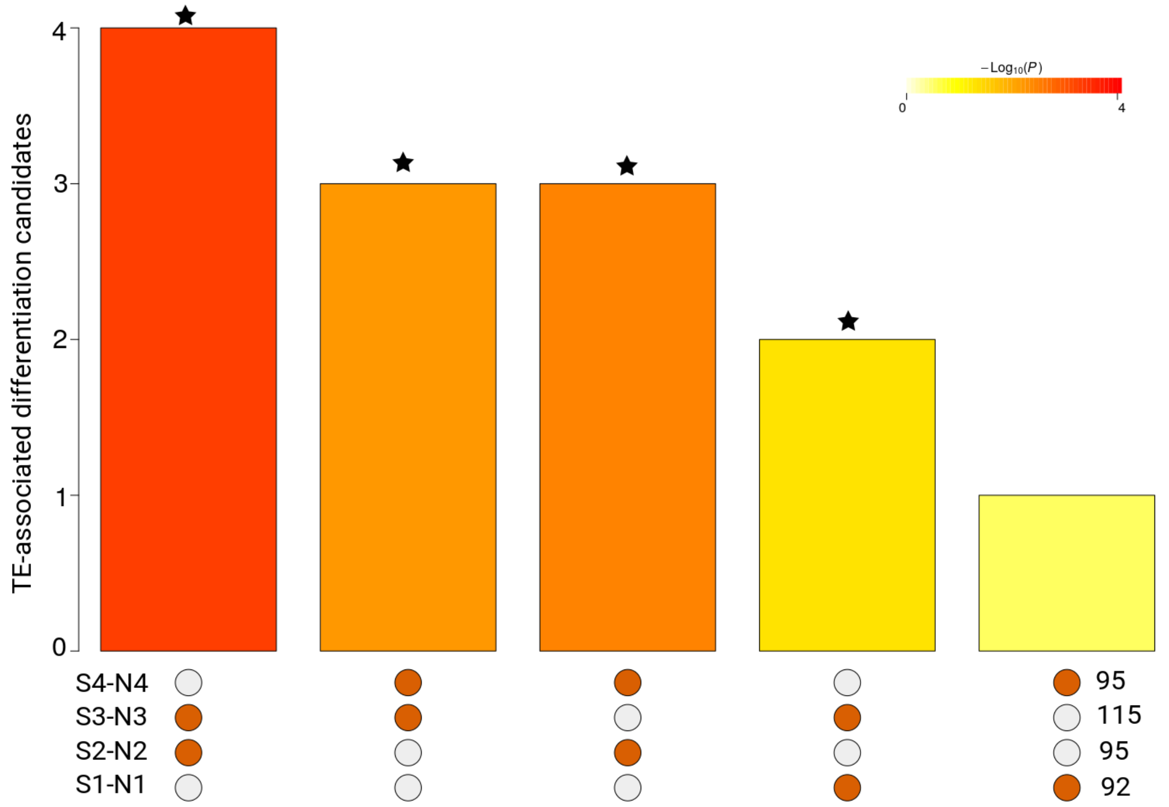

Supplementary Fig. 9. Intersection among lists of TE-associated candidates (1% empirical F_{ST} outliers) within each population pair (P1-P4); significant (p < 0.05) overlaps are marked with asterisks; the results of one-sided Fisher's exact test are available in Supplementary Data 10.

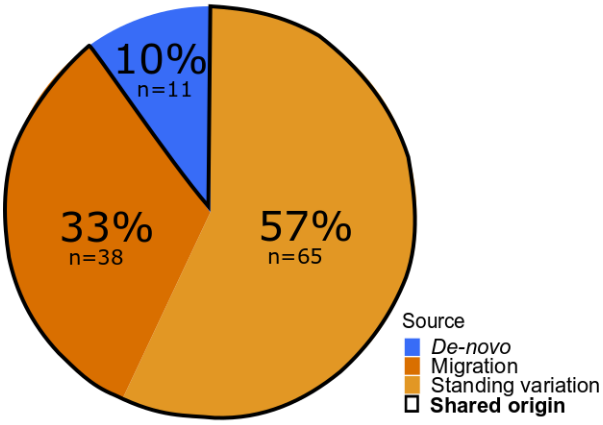

Supplementary Fig. 10. Sources of variation inferred by DMC analysis of the 246 serpentine adaptation candidates that were identified by applying less stringent differentiation threshold (upper 3 % differentiation outlier threshold). The candidates were further overlapped across population pairs leading to 1179 parallel differentiation candidates and further by 2809 LFMM candidates. Pie chart summarizes the proportions of cases of parallel adaptation (114 significantly non-neutral cases in total) reflecting likely origin from from *de-novo* mutations, shared from standing variation and by gene flow. The statistics for individual candidate loci are summarized in Supplementary Data 11.

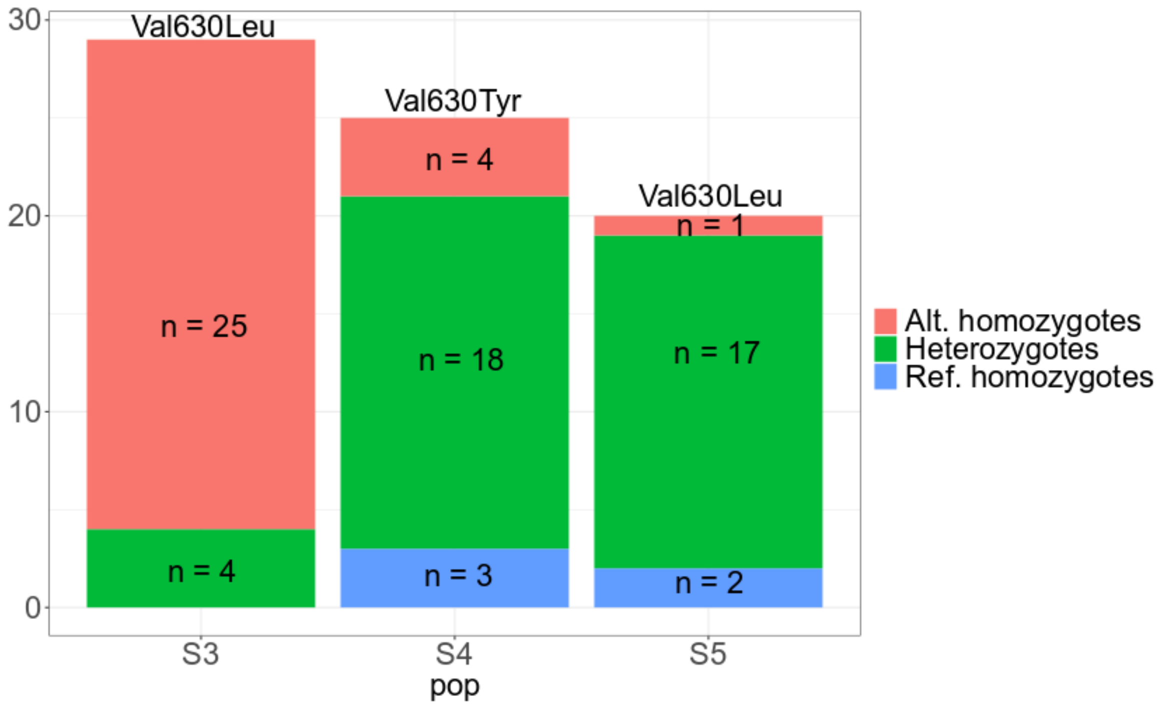

Supplementary Fig. 11. Genotype frequencies in residue 630 in the *TPC1* locus in the three serpentine *A. arenosa* populations encompassing serpentine-specific variants at this position. Alternative homozygous state (Alt. homozygotes) is represented by private allele C (mutation Val630Leu) in S3 and S5 populations and another private allele T in S4 population (mutation Val630Tyr). Heterozygous state is represented by alleles C/G in S3 population, A/C/G alleles in S5 population, and T/G in S4 population. Reference homozygous state (Ref. homozygotes) is represented by the widespread non-serpentine allele G (Val630Val).

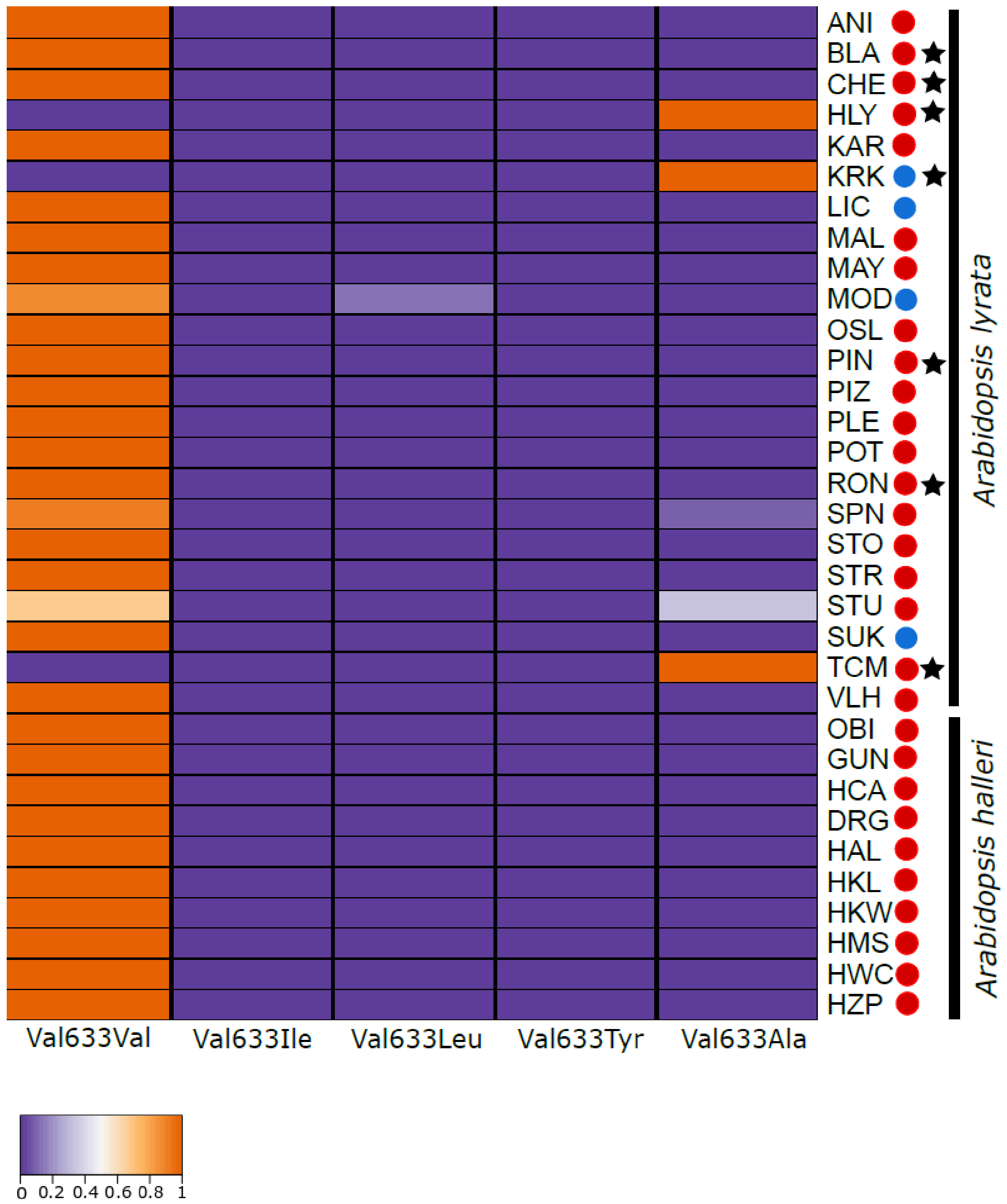

Supplementary Fig. 12. Allele frequencies of amino acid substitutions (colour key) in *A. lyrata* residue 633 (orthologous to 630 in *A. arenosa*) of the *TPC1* locus in *A. halleri* and *A. lyrata* populations inferred by a reanalysis of the available short read data. Red points: diploids, blue points: tetraploids. The substitution of Val633Leu found rarely in one *A. lyrata* population (MOD, limestone population) is encoded by a different codon (GTA -> TTA) than in *A. arenosa* serpentine populations (GTA -> CTA) and is thus non-homologous. “Population” samples represented by only one individual are marked by a star. On X-axis are the amino acid substitutions accompanied with the ancestral state Val633Val and on Y-axis are population codes for *A. halleri* and *A. lyrata* populations[10–16].

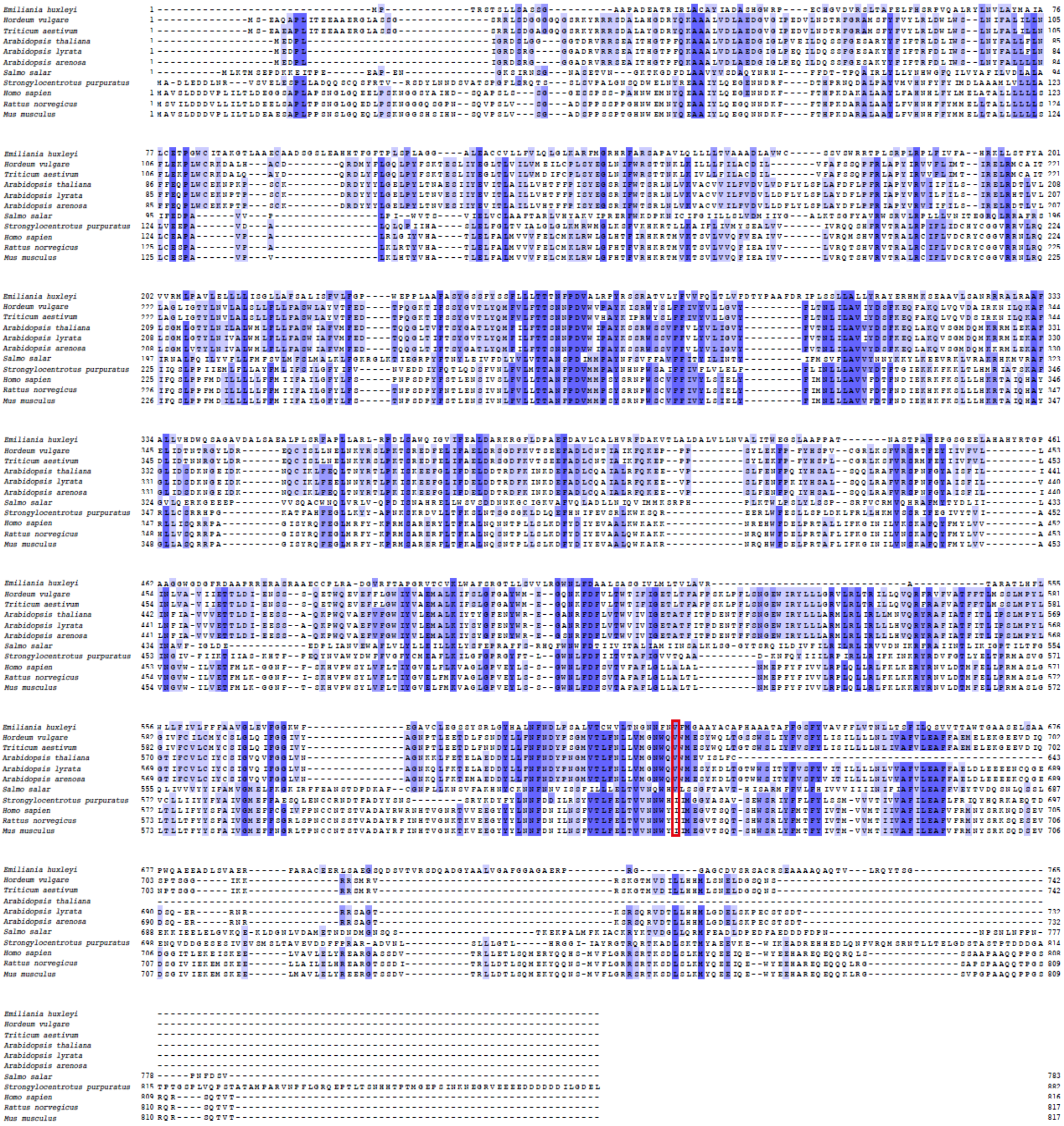

Supplementary Fig. 13. Cross-kingdom sequence conservation of the *TPC1* locus. Multiple sequence alignment of *TPC1* sequences (*Emiliania huxleyi, Hordeum vulgare, Arabidopsis thaliana, Triticum aestivum, Salmo salar, Strongylocentrotus purpuratus, Homo sapiens, Rattus norvegicus* and *Mus musculus*). Residues are coloured according to the percentage that match the consensus sequence from 100% (dark blue) to 0% (white). The position of the serpentine – specific high frequency non-synonymous SNP (Val634 in *A. thaliana*, Val630 in *A. arenosa* and Val633 in *A. lyrata*) is highlighted in red. Non-serpentine consensus sequences are shown for the three *Arabidopsis* species.

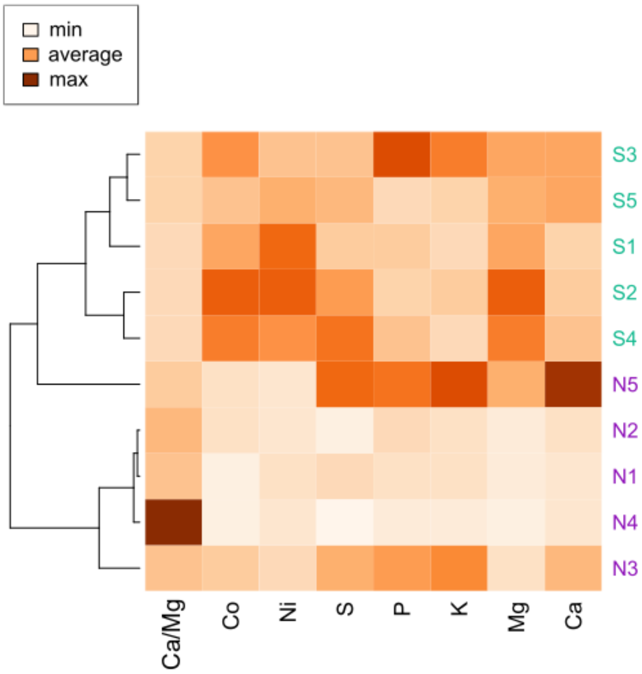

Supplementary Fig. 14. Heatmap of population clustering (UPGMA) based on particular soil elemental concentrations. Note: the elemental concentrations were centred and scaled using heatmap function in R scale='column' (for the original values see Supplementary Table 4).

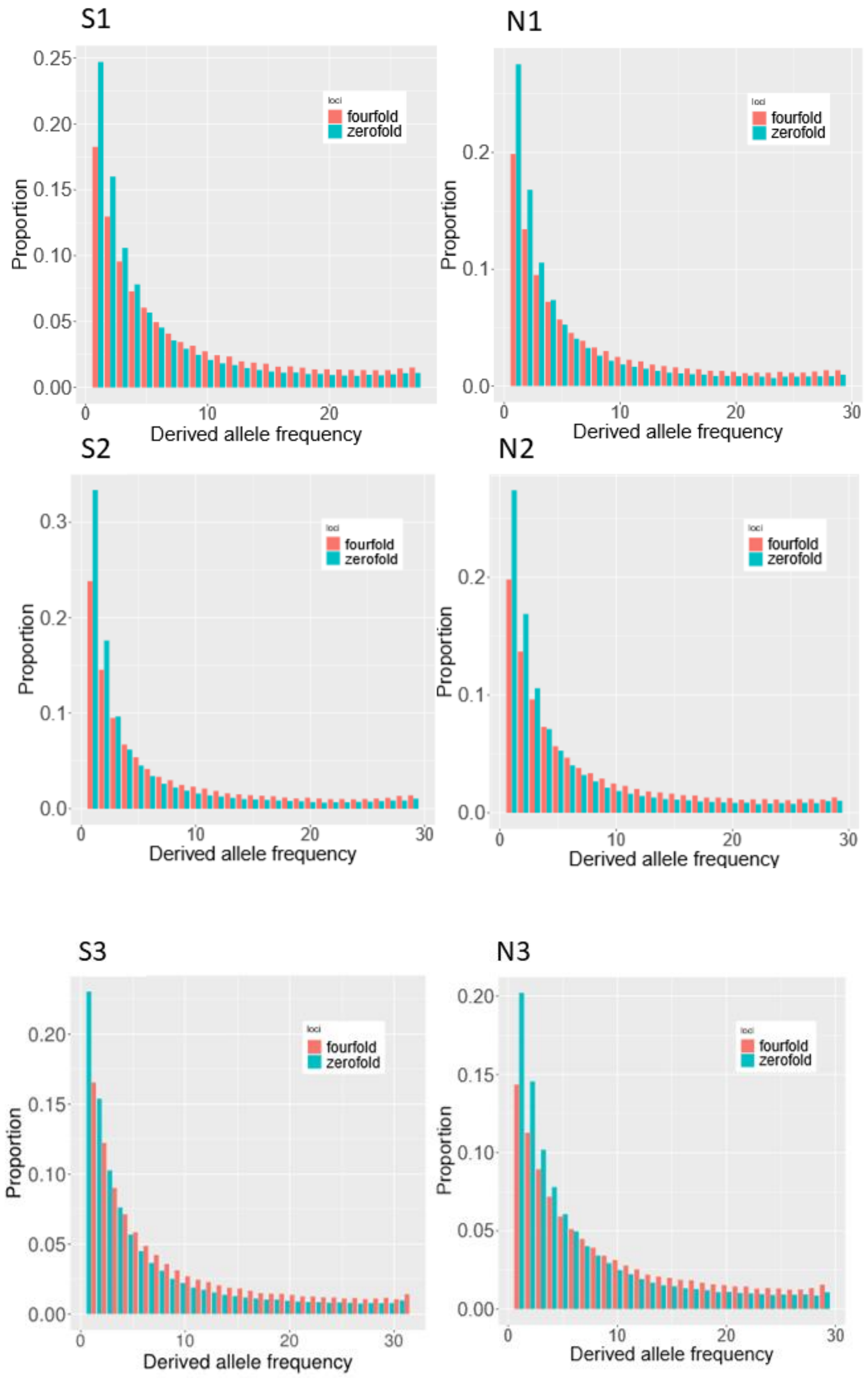
S1
N1
S2
N2
S3
N3
Proportion
Derived allele frequency
loci
fourfold
zerofold
0.25
0.20
0.15
0.10
0.05
0.00
0.0
0.1
0.2
0.3
0
10
20
30

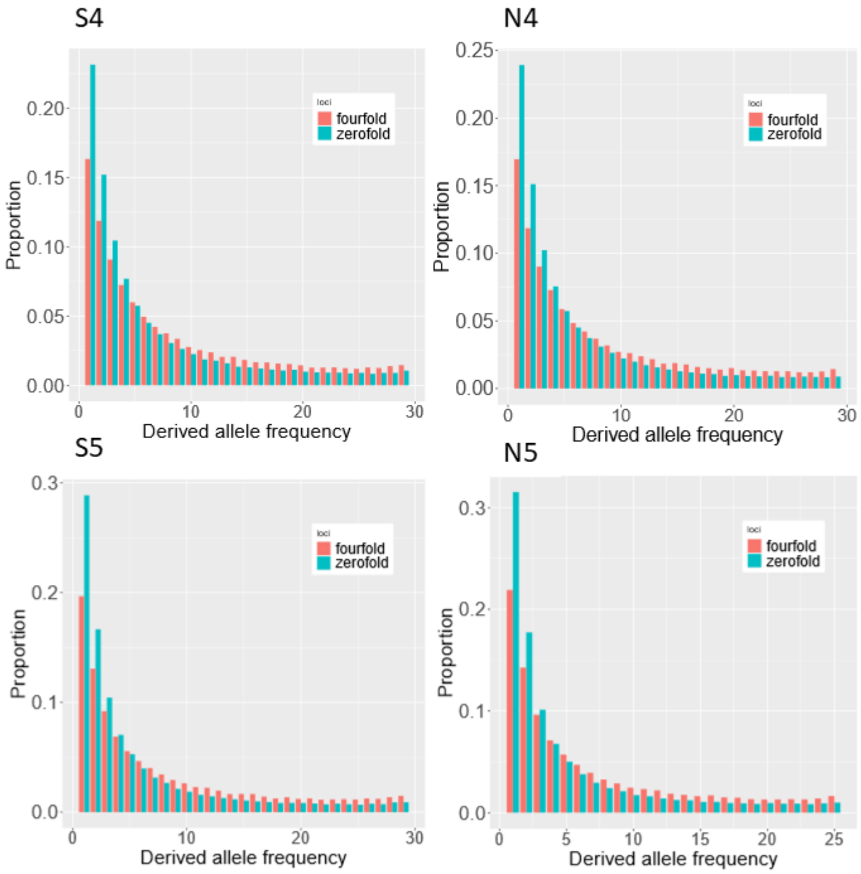

Supplementary Fig. 15. Site frequency spectra of fourfold and zerofold sites for all studied populations. Figure shows excess of singletons in zerofold sites suggesting genome-wide purifying selection in line with previous analyses in *A. arenosa*[7,17]. Note: based on the level of missingness we downsampled the data to the average number of observed alleles per particular site (32 alleles: pop S3; 30 alleles: N1, S2, N2, N3, S4, N4, and S5; 28 alleles: S1; 26 alleles: N5); sites with fixed alleles are not shown.

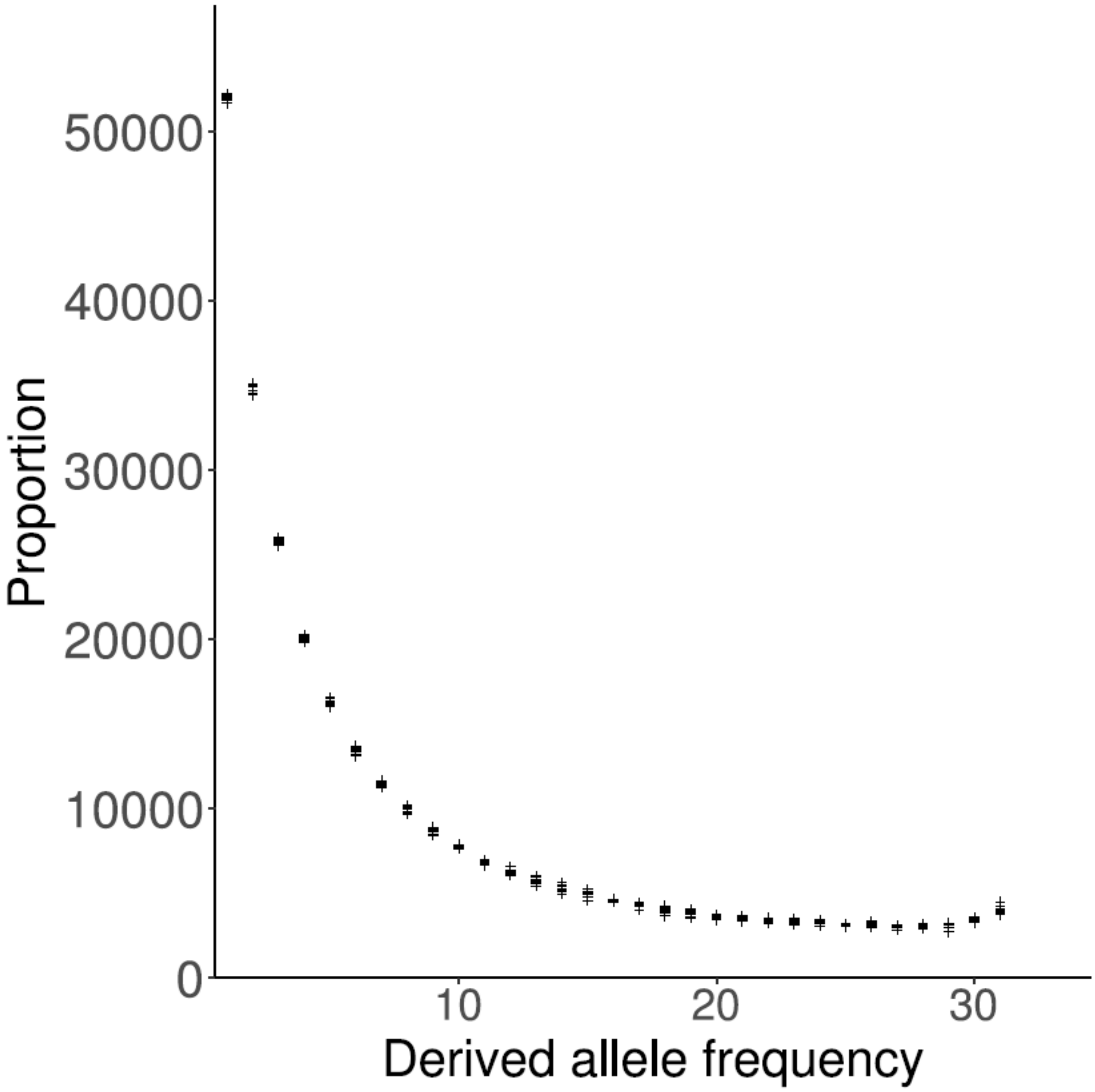

Supplementary Fig. 16. Site frequency spectrum from 10 iterations of random subsampling to 8 individuals (i.e. 32 alleles) from all available 17 individuals of S3 population (including the data from Arnold et al.[5]). Y shows proportion of sites with an allele of given frequency X, separately for each iteration by a horizontal line. Very narrow range of the estimates implies that putative sampling and coverage bias has negligible effect on the allele frequency estimates summarized in the site frequency spectrum.

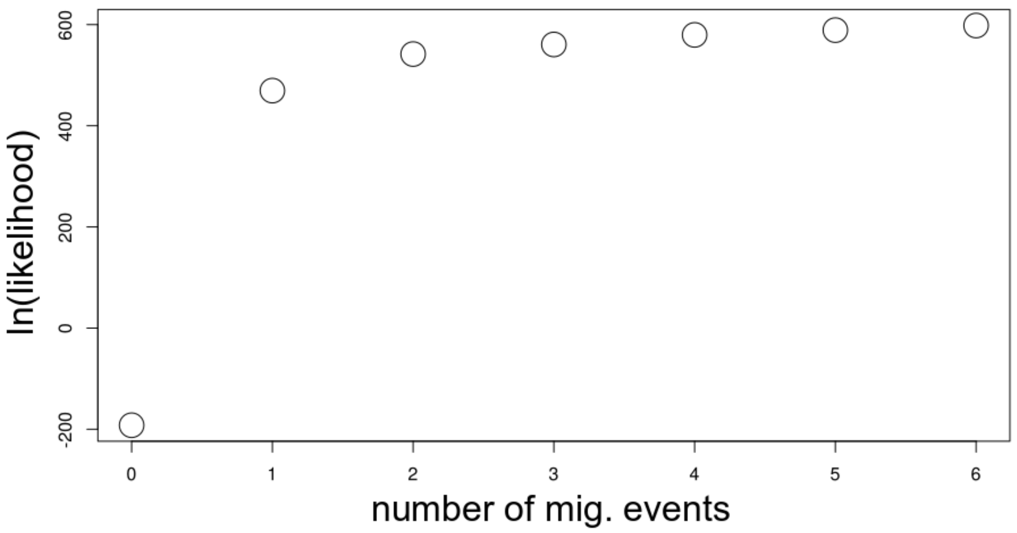

Supplementary Fig. 17. Pattern of increasing likelihood with rising number of migration events from one to six in Treemix analyses, showing saturation after adding one migration event (from outgroup to the node with N3 population).

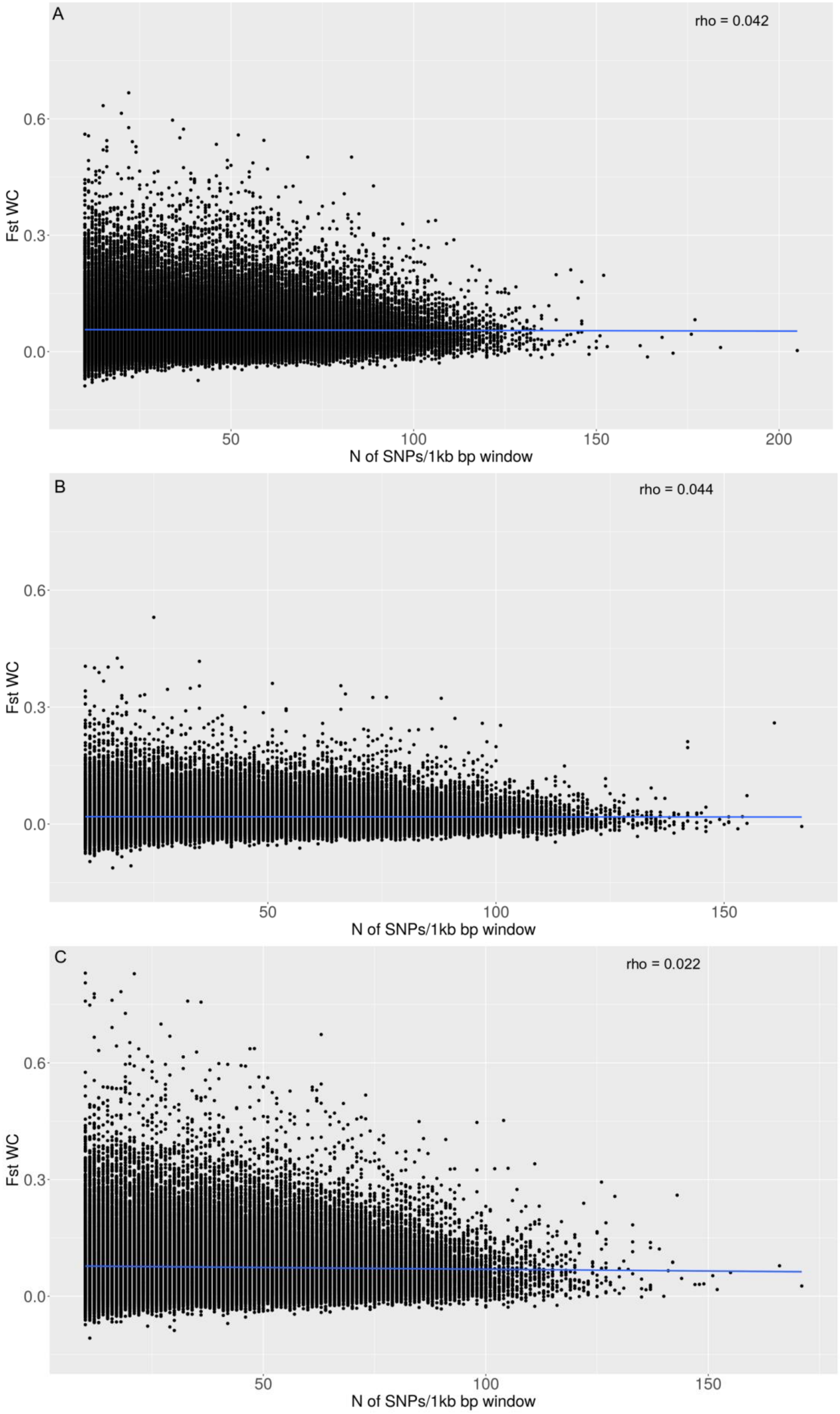
A
rho = 0.042
Fst WC
0.6
0.3
0.0
50
100
150
200
N of SNPs/1kb bp window
B
rho = 0.044
Fst WC
0.6
0.3
0.0
50
100
150
N of SNPs/1kb bp window
C
rho = 0.022
Fst WC
0.6
0.3
0.0
50
100
150
N of SNPs/1kb bp window

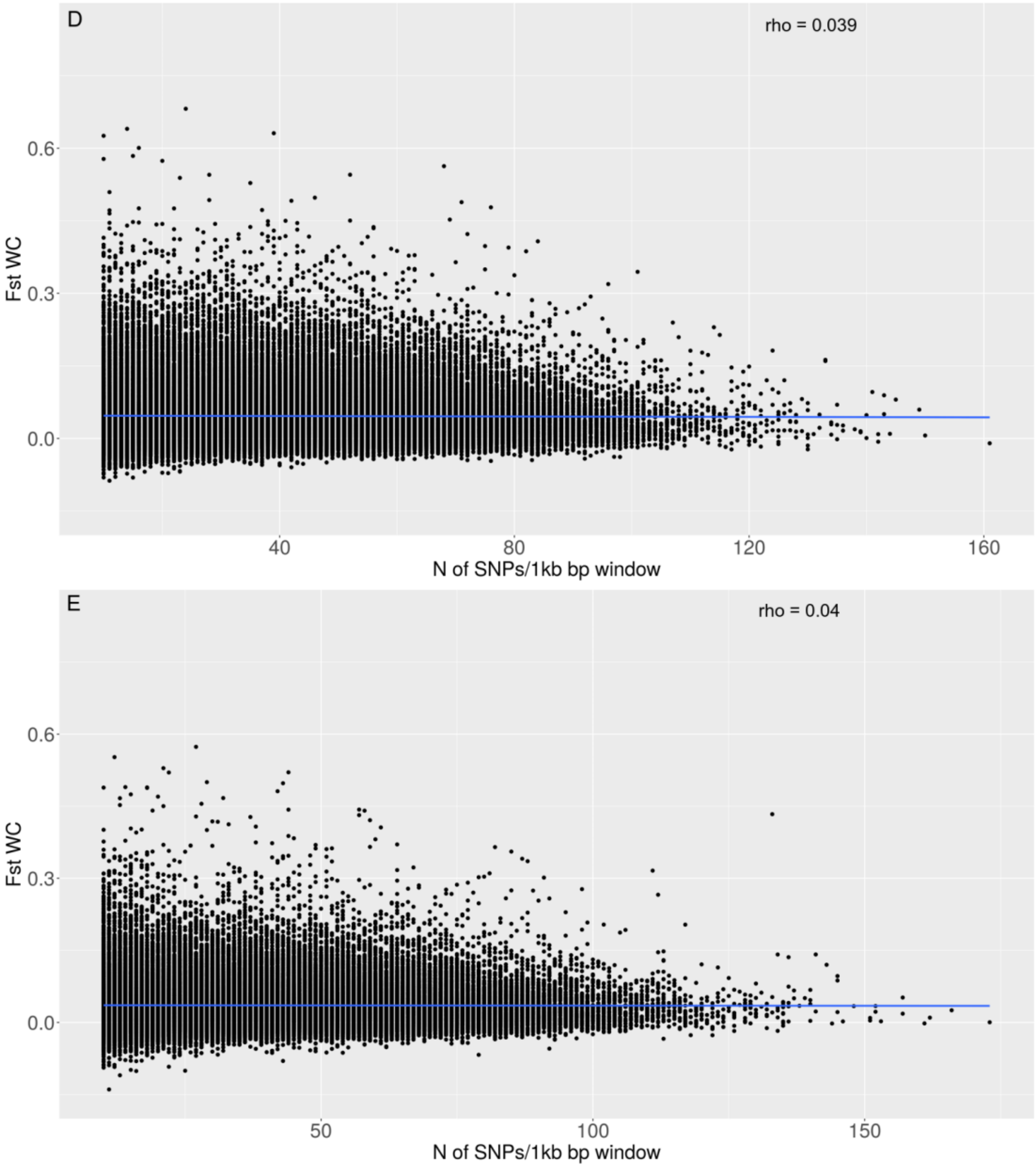

Supplementary Fig. 18. Relationships between F_{ST} estimates for each 1kbp window and the number of SNPs per window for each population pair 1 - 5 (a-e). Note: low-informative windows with <10 SNPs were excluded; rho = Spearman's rank correlation coefficient; the regression line is in blue.

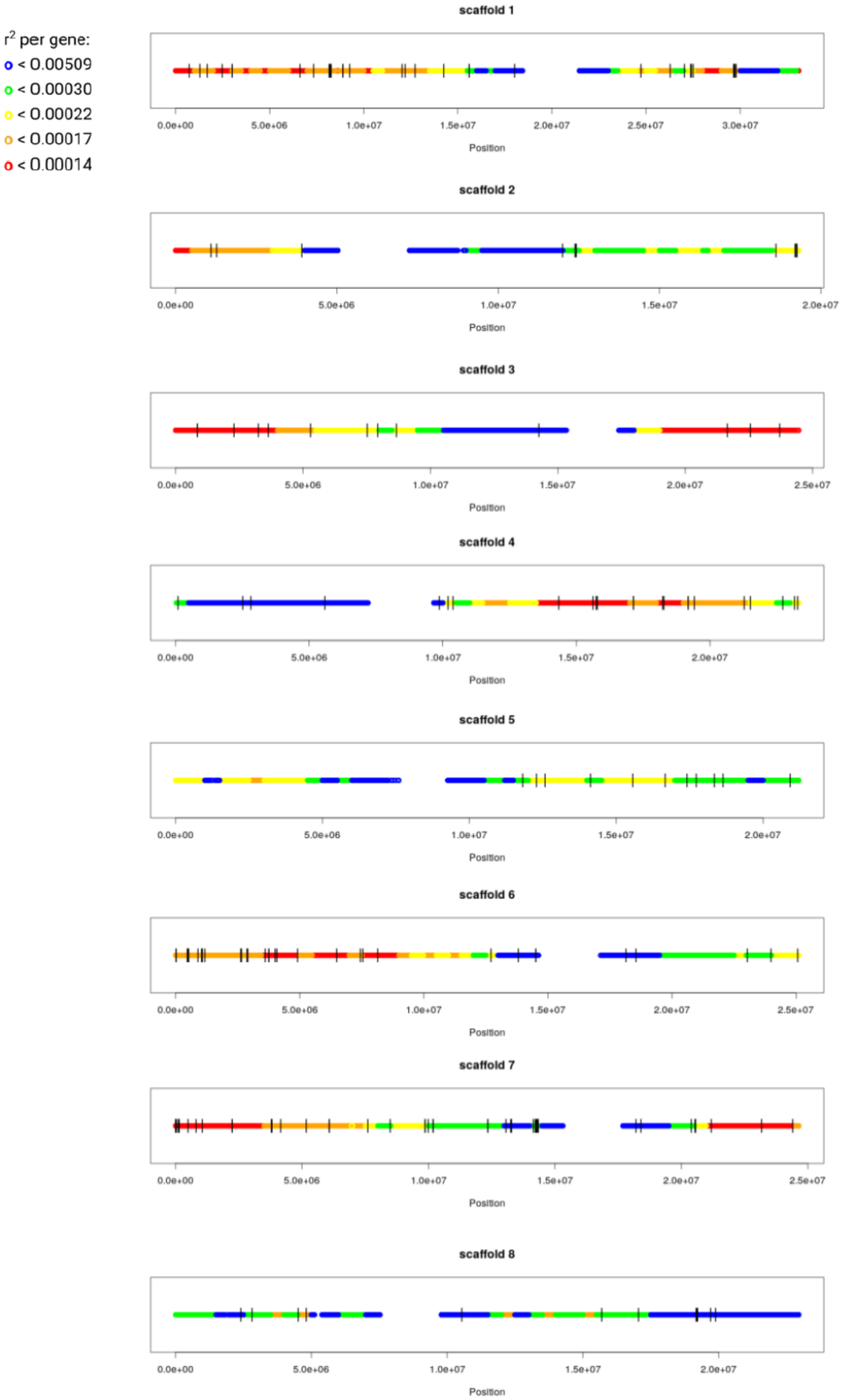

Supplementary Fig. 19. Location of all parallel differentiation candidates (black vertical lines) on *A. lyrata* reference chromosomes colored by bins of distinct recombination rate per gene as estimated based on the available *A. lyrata* genetic map[8]. The figure illustrates that parallel differentiation candidates are distributed throughout the genome, not being limited to regions with low recombination rate per gene (red/orange).

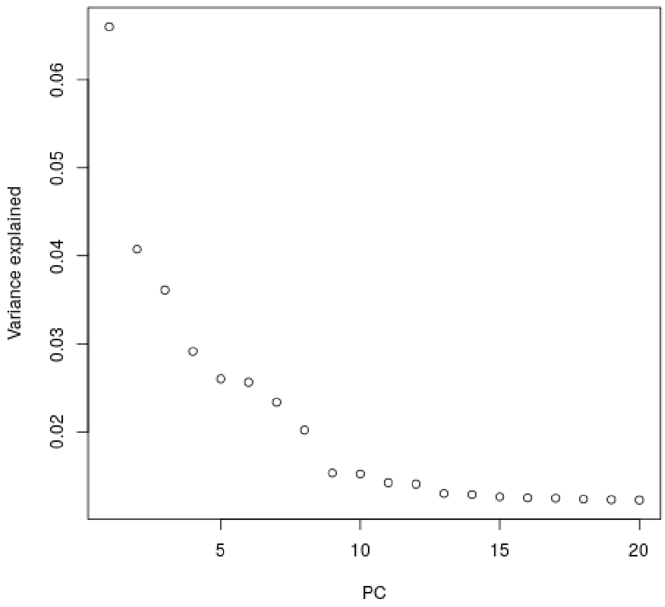

Supplementary Fig. 20. Decreasing proportion of explained variation by principal components (PC) of a PCA based on ~1 M 4dg SNPs indicating considerable improvement of explained variation up to five main PCs.

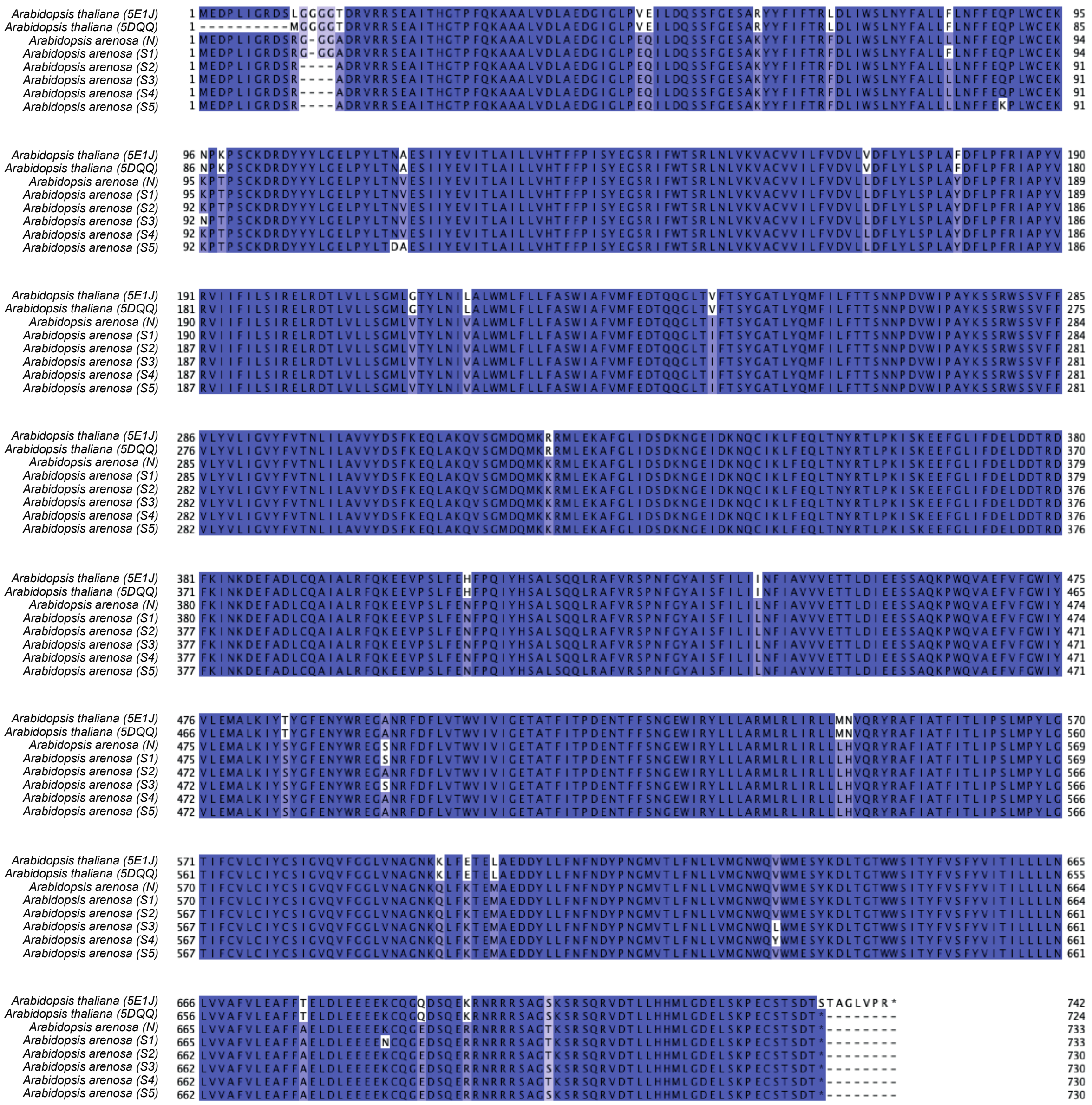

Supplementary Fig. 21. Multiple sequence alignment of *TPC1* consensus sequences used for structural homology modelling*.* Shown are consensus sequences from non-serpentine *A. arenosa* (N), five serpentine *A. arenosa,* and two non-serpentine *A. thaliana* crystal structures (5E1J and 5DQQ[18,19]). Residues are coloured according to percentage identity from 100% (dark blue) to 0% (white). Residue 630 in *A. arenosa* (634 in *A. thaliana* and 633 in *A. lyrata)* where the only serpentine-specific variation occurs is highlighted in red frame. The eight other nonsynonymous polymorphisms showing within *A. arenosa* variation are not serpentine specific (Supplementary Table 7) and sit away from any known functional sites, indicating that they are likely non-functional (Fig. 3d).

Supplementary references

1. Danku, J. M. C., Lahner, B., Yakubova, E. & Salt, D. E. Large-scale plant ionomics. *Methods Mol. Biol.* **953**, 255–276 (2013).
2. Turner, T. L., Bourne, E. C., Von Wettberg, E. J., Hu, T. T. & Nuzhdin, S. V. Population resequencing reveals local adaptation of *Arabidopsis lyrata* to serpentine soils. *Nat. Genet.* **42**, 260–263 (2010).
3. Sobczyk, M. K., Smith, J. A. C., Pollard, A. J. & Filatov, D. A. Evolution of nickel hyperaccumulation and serpentine adaptation in the *Alyssum serpyllifolium* species complex. *Heredity (Edinb).* **118**, 31–41 (2017).
4. Selby, J. P. The genetic basis of local adaptation to serpentine soils in *Mimulus guttatus*. *Doctoral dissertation, Duke University* (2014).
5. Arnold, B. J. *et al.* Borrowed alleles and convergence in serpentine adaptation. *Proc. Natl. Acad. Sci.* **113**, 8320–8325 (2016).
6. Monnahan, P. *et al.* Pervasive population genomic consequences of genome duplication in *Arabidopsis arenosa*. *Nat. Ecol. Evol.* **3**, 457 (2019).
7. Arnold, B., Kim, S. T. & Bomblies, K. Single geographic origin of a widespread autotetraploid *Arabidopsis arenosa* lineage followed by interploidy admixture. *Mol. Biol. Evol.* **32**, 1382–1395 (2015).
8. Hämälä, T. & Savolainen, O. Genomic patterns of local adaptation under gene flow in *Arabidopsis lyrata*. *Mol. Biol. Evol.* **36**, 2557–2571 (2019).
9. Weir, B. S. & Cockerham, C. C. Estimating F-statistics for the analysis of population structure. *Evolution.* **38**, 1358–1370 (1984).
10. Novikova, P. Y. *et al.* Sequencing of the genus *Arabidopsis* identifies a complex history of nonbifurcating speciation and abundant trans-specific polymorphism. *Nat. Genet.* **48**, 1077–1082 (2016).
11. Hämälä, T., Mattila, T. M., Leinonen, P. H., Kuittinen, H. & Savolainen, O. Role of seed germination in adaptation and reproductive isolation in *Arabidopsis lyrata*. *Mol. Ecol.* **26**, 3484–3496 (2017).
12. Marburger, S. *et al.* Interspecific introgression mediates adaptation to whole genome duplication. *Nat. Commun.* **10**, 1–11 (2019).
13. Guggisberg, A. *et al.* The genomic basis of adaptation to calcareous and siliceous soils in *Arabidopsis lyrata*. *Mol. Ecol.* **27**, 5088–5103 (2018).
14. Mattila, T. M., Tyrmi, J., Pyhäjärvi, T. & Savolainen, O. Genome-wide analysis of colonization history and concomitant selection in *Arabidopsis lyrata*. *Mol. Biol. Evol.* **34**, 2665–2677 (2017).
15. Preite, V. *et al.* Convergent evolution in *Arabidopsis halleri* and *Arabidopsis arenosa* on calamine metalliferous soils. *Philos. Trans. R. Soc. B* **374**, 20180243 (2019).
16. Bohutínská, M. *et al.* Genomic basis of parallel adaptation varies with divergence in

Arabidopsis and its relatives. *Proc. Natl. Acad. Sci.* **118**, e2022713118 (2021).

17. Hollister, J. D. *et al.* Genetic adaptation associated with genome-doubling in autotetraploid *Arabidopsis arenosa*. *PLoS Genet.* **8**, e1003093 (2012).

18. Guo, J. *et al.* Structure of the voltage-gated two-pore channel *TPC1* from *Arabidopsis thaliana*. *Nature* **531**, 196–201 (2016).

19. Kintzer, A. F. & Stroud, R. M. Structure, inhibition and regulation of two-pore channel *TPC1* from *Arabidopsis thaliana*. *Nature* **531**, 258–262 (2016).
