# Supplementary material for: Parallel adaptation in autopolyploid Arabidopsis arenosa is dominated by repeated recruitment of shared alleles
Source: Nat Commun. 2021 Aug 17;12:4979. doi: 10.1038/s41467-021-25256-5 (PMC8370997; doi:10.1038/s41467-021-25256-5)
Supplement: Supplementary file 3 — Supplementary Data 1-12 [file 41467_2021_25256_MOESM3_ESM.zip › Supplemementary_data/Supplementary_data_12.docx]

Supplementary Dataset 12: Examples of parameter files used in *fastsimcoal2* simulations to test for parallel origin of serpentine populations.

1A) Parallel origin *.tpl file

//Parameters for the coalescence simulation program: simcoal.exe

4 samples to simulate:

//Population effective sizes (number of genes), order as in the input DSFS, below in order 0 1 2

N_POP0

N_POP1

N_POP2

N_POP3

//Samples sizes and samples age (in Nchr)

0

0

0

0

//Growth rates: negative growth implies population expansion

0

0

0

0

//Number of migration matrices: 0 implies no migration between demes (bakwards in time, i.e. target merging with source)

2

//Migration matrix 0

0 0 0 0

0 0 0 0

0 0 0 0

0 0 0 0

//Migration matrix 1

0 0 0 0

0 0 0 0

0 0 0 0

0 0 0 0

//historical event: time, source, sink, migrants, new deme size, new growth rate, migration matrix index

3 historical event

TDIV1 0 1 1 ResizeTIME1 0 1

TDIV2 2 3 1 ResizeTIME2 0 1

TDIV3 1 3 1 ResizeTIME3 0 1

//Number of independent loci [chromosome]

1 0

//Per chromosome: Number of contiguous linkage Block: a block is a set of contiguous loci

1

//per Block:data type, number of loci, per generation recombination and mutation rates and optional parameters

FREQ 1 0 4.3e-8 OUTEXP

1B) Parallel origin *.est file

// Priors and rules file

// *********************

[PARAMETERS]

//all N are in number of haploid individuals

1 N_POP0 unif 10 1e6 output

1 N_POP1 unif 10 1e6 output

1 N_POP2 unif 10 1e6 output

1 N_POP3 unif 10 1e6 output

1 N_ANCAll unif 10 1e7 output

1 N_ANC01 unif 10 2e6 output

1 N_ANC23 unif 10 2e6 output

1 TDIV1 unif 10 1e4 output

1 TDIV2 unif 10 1e4 output

1 TPLUS unif 10 3e4 hide

[RULES]

[COMPLEX PARAMETERS]

1 TDIVMAX = TDIV1 %max% TDIV2 hide

1 TDIV3 = TDIVMAX+TPLUS ouput

0 ResizeTIME1 = N_ANC01/N_POP1 hide

0 ResizeTIME2 = N_ANC23/N_POP3 hide

0 ResizeTIME3 = N_ANCAll/N_ANC23 hide

2A) Parallel origin – migration *.tpl file

//Parameters for the coalescence simulation program: simcoal.exe

4 samples to simulate:

//Population effective sizes (number of genes), order as in the input DSFS, below in order 0 1 2

N_POP0

N_POP1

N_POP2

N_POP3

//Samples sizes and samples age (in Nchr)

0

0

0

0

//Growth rates: negative growth implies population expansion

0

0

0

0

//Number of migration matrices: 0 implies no migration between demes (bakwards in time, i.e. target merging with source)

2

//Migration matrix 0

0 Mig01 0 0

Mig10 0 0 0

0 0 0 Mig23

0 0 Mig32 0

//Migration matrix 1

0 0 0 0

0 0 0 0

0 0 0 0

0 0 0 0

//historical event: time, source, sink, migrants, new deme size, new growth rate, migration matrix index

3 historical event

TDIV1 0 1 1 ResizeTIME1 0 1

TDIV2 2 3 1 ResizeTIME2 0 1

TDIV3 1 3 1 ResizeTIME3 0 1

//Number of independent loci [chromosome]

1 0

//Per chromosome: Number of contiguous linkage Block: a block is a set of contiguous loci

1

//per Block:data type, number of loci, per generation recombination and mutation rates and optional parameters

FREQ 1 0 4.3e-8 OUTEXP

2B) Parallel origin – migration *.est file

// Priors and rules file

// *********************

[PARAMETERS]

//all N are in number of haploid individuals

1 N_POP0 unif 10 1e6 output

1 N_POP1 unif 10 1e6 output

1 N_POP2 unif 10 1e6 output

1 N_POP3 unif 10 1e6 output

1 N_ANCAll unif 10 1e7 output

1 N_ANC01 unif 10 2e6 output

1 N_ANC23 unif 10 2e6 output

1 TDIV1 unif 10 1e4 output

1 TDIV2 unif 10 1e4 output

1 TPLUS unif 10 3e4 hide

0 Mig01 logunif 1e-7 1e-5 output

0 Mig10 logunif 1e-7 1e-5 output

0 Mig23 logunif 1e-7 1e-5 output

0 Mig32 logunif 1e-7 1e-5 output

[RULES]

[COMPLEX PARAMETERS]

1 TDIVMAX = TDIV1 %max% TDIV2 hide

1 TDIV3 = TDIVMAX+TPLUS ouput

0 ResizeTIME1 = N_ANC01/N_POP1 hide

0 ResizeTIME2 = N_ANC23/N_POP3 hide

0 ResizeTIME3 = N_ANCAll/N_ANC23 hide

3A) Single origin *.tpl file

//Parameters for the coalescence simulation program: simcoal.exe

4 samples to simulate:

//Population effective sizes (number of genes), order as in the input DSFS, below in order 0 1 2

N_POP0

N_POP1

N_POP2

N_POP3

//Samples sizes and samples age (in Nchr)

0

0

0

0

//Growth rates: negative growth implies population expansion

0

0

0

0

//Number of migration matrices: 0 implies no migration between demes (bakwards in time, i.e. target merging with source)

2

//Migration matrix 0

0 0 0 0

0 0 0 0

0 0 0 0

0 0 0 0

//Migration matrix 1

0 0 0 0

0 0 0 0

0 0 0 0

0 0 0 0

//historical event: time, source, sink, migrants, new deme size, new growth rate, migration matrix index

3 historical event

TDIV1 0 1 1 ResizeTIME1 0 1

TDIV2 2 3 1 ResizeTIME2 0 1

TDIV3 1 3 1 ResizeTIME3 0 1

//Number of independent loci [chromosome]

1 0

//Per chromosome: Number of contiguous linkage Block: a block is a set of contiguous loci

1

//per Block:data type, number of loci, per generation recombination and mutation rates and optional parameters

FREQ 1 0 4.3e-8 OUTEXP

3B) Single origin *.est file

// Priors and rules file

// *********************

[PARAMETERS]

//#isInt? #name #dist.#min #max

//all N are in number of haploid individuals

1 N_POP0 unif 10 1e6 output

1 N_POP1 unif 10 1e6 output

1 N_POP2 unif 10 1e6 output

1 N_POP3 unif 10 1e6 output

1 N_ANCAll unif 10 1e7 output

1 N_ANC01 unif 10 2e6 output

1 N_ANC23 unif 10 2e6 output

1 TDIV1 unif 10 1e4 output

1 TDIV2 unif 10 1e4 output

1 TPLUS unif 10 3e4 hide

[RULES]

[COMPLEX PARAMETERS]

1 TDIVMAX = TDIV1 %max% TDIV2 hide

1 TDIV3 = TDIVMAX+TPLUS ouput

0 ResizeTIME1 = N_ANC01/N_POP1 hide

0 ResizeTIME2 = N_ANC23/N_POP3 hide

0 ResizeTIME3 = N_ANCAll/N_ANC23 hide

4A) Single origin -migration *.tpl file

//Parameters for the coalescence simulation program: simcoal.exe

4 samples to simulate:

//Population effective sizes (number of genes), order as in the input DSFS, below in order 0 1 2

N_POP0

N_POP1

N_POP2

N_POP3

//Samples sizes and samples age (in Nchr)

0

0

0

0

//Growth rates: negative growth implies population expansion

0

0

0

0

//Number of migration matrices: 0 implies no migration between demes (bakwards in time, i.e. target merging with source)

2

//Migration matrix 0

0 0 Mig02 0

0 0 0 Mig13

Mig20 0 0 0

0 Mig31 0 0

//Migration matrix 1

0 0 0 0

0 0 0 0

0 0 0 0

0 0 0 0

//historical event: time, source, sink, migrants, new deme size, new growth rate, migration matrix index

3 historical event

TDIV1 0 1 1 ResizeTIME1 0 1

TDIV2 2 3 1 ResizeTIME2 0 1

TDIV3 1 3 1 ResizeTIME3 0 1

//Number of independent loci [chromosome]

1 0

//Per chromosome: Number of contiguous linkage Block: a block is a set of contiguous loci

1

//per Block:data type, number of loci, per generation recombination and mutation rates and optional parameters

FREQ 1 0 4.3e-8 OUTEXP

4A) Single origin -migration *.est file

// Priors and rules file

// *********************

[PARAMETERS]

//all N are in number of haploid individuals

1 N_POP0 unif 10 1e6 output

1 N_POP1 unif 10 1e6 output

1 N_POP2 unif 10 1e6 output

1 N_POP3 unif 10 1e6 output

1 N_ANCAll unif 10 1e7 output

1 N_ANC01 unif 10 2e6 output

1 N_ANC23 unif 10 2e6 output

1 TDIV1 unif 10 1e4 output

1 TDIV2 unif 10 1e4 output

1 TPLUS unif 10 3e4 hide

0 Mig02 logunif 1e-7 1e-5 output

0 Mig13 logunif 1e-7 1e-5 output

0 Mig20 logunif 1e-7 1e-5 output

0 Mig31 logunif 1e-7 1e-5 output

[RULES]

[COMPLEX PARAMETERS]

1 TDIVMAX = TDIV1 %max% TDIV2 hide

1 TDIV3 = TDIVMAX+TPLUS ouput

0 ResizeTIME1 = N_ANC01/N_POP1 hide

0 ResizeTIME2 = N_ANC23/N_POP3 hide

0 ResizeTIME3 = N_ANCAll/N_ANC23 hide
