# Supplementary material for: Parallel adaptation in autopolyploid Arabidopsis arenosa is dominated by repeated recruitment of shared alleles
Source: Nat Commun. 2021 Aug 17;12:4979. doi: 10.1038/s41467-021-25256-5 (PMC8370997; doi:10.1038/s41467-021-25256-5)
Supplement: Supplementary file 4 — Description of Additional Supplementary Files [file 41467_2021_25256_MOESM4_ESM.pdf]

## Description of additional supplementary information

Title: Supplementary Data 1

Description: Sequence processing and quality assessment of each re-sequenced individual.

Title: Supplementary Data 2

Description: Scenario selection results from fastsimcoal for each population quartet and simulated scenario.

Title: Supplementary Data 3

Description: Description of differentiation candidates (1% of empirical FST outliers) for 5 pairs.

Title: Supplementary Data 4

Description: Results from GO enrichment analysis of the union of all 2245 differentiation candidates and of 207 parallel differentiation candidates.

Title: Supplementary Data 5

Description: Description of genes with at least one SNP associated with soil content of at least one element (Ca/Mg, Co, Mg and Ni) across all samples.

Title: Supplementary Data 6

Description: Fisher exact test on overlap between parallel differentiation candidates and LFMM candidates.

Title: Supplementary Data 7

Description: Summary of 61 serpentine adaptation candidates with identification of the variation source and their GO enrichment analysis.

Supplementary Data 8

Description: Summary of TE variants.

Title: Supplementary Data 9

Description: Results from GO enrichment analysis of differentiation TE candidate genes.

Title: Supplementary Data 10

Description: Summary of 246 serpentine adaptation candidates with identification of the variation source in DMC.

Title: Supplementary Data 11

Description: Examples of parameter files used in fastsimcoal2 simulations to test for parallel origin of serpentine populations.

Title: Supplementary Data 12

Description: Examples of parameter files used in fastsimcoal2 simulations to test for parallel origin of serpentine populations.
