# Supplementary material for: Parallel adaptation in autopolyploid Arabidopsis arenosa is dominated by repeated recruitment of shared alleles
Source: Nat Commun. 2021 Aug 17;12:4979. doi: 10.1038/s41467-021-25256-5 (PMC8370997; doi:10.1038/s41467-021-25256-5)
Supplement: Supplementary file 5 — Reporting Summary [file 41467_2021_25256_MOESM5_ESM.pdf]

nature research

Corresponding author(s): Filip Kolar

Last updated by author(s): Jul 8, 2021

Reporting Summary

Nature Research wishes to improve the reproducibility of the work that we publish. This form provides structure for consistency and transparency in reporting. For further information on Nature Research policies, see our Editorial Policies and the Editorial Policy Checklist.

Statistics

For all statistical analyses, confirm that the following items are present in the figure legend, table legend, main text, or Methods section.

n/a	Confirmed	
☐	☒	The exact sample size (*n*) for each experimental group/condition, given as a discrete number and unit of measurement
☐	☒	A statement on whether measurements were taken from distinct samples or whether the same sample was measured repeatedly
☐	☒	The statistical test(s) used AND whether they are one- or two-sided<br>*Only common tests should be described solely by name; describe more complex techniques in the Methods section.*
☒	☐	A description of all covariates tested
☐	☒	A description of any assumptions or corrections, such as tests of normality and adjustment for multiple comparisons
☐	☒	A full description of the statistical parameters including central tendency (e.g. means) or other basic estimates (e.g. regression coefficient) AND variation (e.g. standard deviation) or associated estimates of uncertainty (e.g. confidence intervals)
☐	☒	For null hypothesis testing, the test statistic (e.g. *F*, *t*, *r*) with confidence intervals, effect sizes, degrees of freedom and *P* value noted<br>*Give P values as exact values whenever suitable.*
☒	☐	For Bayesian analysis, information on the choice of priors and Markov chain Monte Carlo settings
☒	☐	For hierarchical and complex designs, identification of the appropriate level for tests and full reporting of outcomes
☐	☒	Estimates of effect sizes (e.g. Cohen's *d*, Pearson's *r*), indicating how they were calculated

Our web collection on statistics for biologists contains articles on many of the points above.

Software and code

Policy information about availability of computer code

Data collection	No software was used for data collection
Data analysis	bwa-0.7.15, picard-2.8 , GATK v.3.7, fastStructure v.1.0, adegenet v.2.1.1, TreeMix v.1.13, fastsimcoal v.2.6, LFMM 2, qvalue v.2.2, SnpEff v. 4.3, TEPID v.0.8, topGO v.2.42, Modeller v. 9.24

For manuscripts utilizing custom algorithms or software that are central to the research but not yet described in published literature, software must be made available to editors and reviewers. We strongly encourage code deposition in a community repository (e.g. GitHub). See the Nature Research guidelines for submitting code & software for further information.

Data

Policy information about availability of data

All manuscripts must include a data availability statement. This statement should provide the following information, where applicable:

- Accession codes, unique identifiers, or web links for publicly available datasets
- A list of figures that have associated raw data
- A description of any restrictions on data availability

bioproject PRJNA667586 [https://www.ncbi.nlm.nih.gov/bioproject/PRJNA667586]
bioproject PRJNA325082 [https://www.ncbi.nlm.nih.gov/bioproject/PRJNA325082]

Field-specific reporting

Please select the one below that is the best fit for your research. If you are not sure, read the appropriate sections before making your selection.

☐ Life sciences ☐ Behavioural & social sciences ☒ Ecological, evolutionary & environmental sciences

For a reference copy of the document with all sections, see nature.com/documents/nr-reporting-summary-flat.pdf

Ecological, evolutionary & environmental sciences study design

All studies must disclose on these points even when the disclosure is negative.

Study description	Population-level study in a hirerachical design (pairs of natural populations adapted/non-adapted to serpentine)
Research sample	Individual samples of Arabidopsis arenosa, representing all known serpentine populations (8 individuals per each population) and adjacent non-serpentine populations.
Sampling strategy	The sample size reflects availability of the natural serpentine-adapted populations. We sampled all five serpentine populations of A. arenosa known to date and complemented each by a proximal (< 19 km distant) non-serpentine population. All populations grew in similar vegetation (rocky outcrops in open forests or grasslands). Within each population, eight individuals were sampled along a transect, in regular approx 2 m intervals.
Data collection	The experimental data were sampled by V. Konecna and D. Pozarova. They recorded germination by as the appearance of cotyledon leaves for the period of 20 days (counts). Rosette traits were scored in situ in the experimental greenhouse of Charles University. Rosette diameter was measured and leaf number was counted twice a week for five weeks. For all traits, the values were directly recorded into a laptop.
Timing and spatial scale	Timing: Plant cultivation was run between March and May 2019, i.e., to correspond with native growth period of A. arenosa. We measured the rosette diameter and counted number of leaves twice a week for five weeks until rosette growth reached a plateau. Spatial scale: The experiment covered approx 25 m2 and encompassed three population pairs representing all three major areas occupied by serpentine A. arenosa (pairs 1, 2, and 3).
Data exclusions	No experimental data were excluded. From genomic dataset, one individual per each S2 and N5 populations was excluded due to exceptionally bad data quality (low percentage of mapped reads and low read depth, <10 on average) prior further analyses.
Reproducibility	There were no attempts to reproduce the experiment given its large scale and logistical demands.
Randomization	Filed samples were collected along transects of the natural populations in the filed. The serpentine and non-serpentine populations were paired, based on their spatial proximity (adjacent N and S populations formed a pair). During experiments, the position of the potted samples was randomly swapped twice a week. Thus, no experimental group allocation was necessary during the experiment.
Blinding	No blinding has been applied. Ecological reciprocal transplant experiments have been conducted, in which populations cultivated in their soil of origin (e.g., S1 population in soil from S1 site) serve as the control treatment.

Did the study involve field work? ☒ Yes ☐ No

Field work, collection and transport

Field conditions	All sampled populations were natural. All populations grew in similar vegetation (rocky outcrops in open forests or grasslands) and soil type S: serpentine, N: siliceous to neutral rocks.
Location	The populations have been sampled in adjacent areas of the Czech Republic and northern Austria, covering an area between 49.71 N , 15.08 E and 47.31 N, 15.37 E. Elevation ranged in between 414 and 1750 m a.s.l.
Access & import/export	The samples were collected in compliance with national and international laws within the following EU countries: Czech Republic, Austria.
Disturbance	No disturbances have been caused by the study.

Reporting for specific materials, systems and methods

We require information from authors about some types of materials, experimental systems and methods used in many studies. Here, indicate whether each material, system or method listed is relevant to your study. If you are not sure if a list item applies to your research, read the appropriate section before selecting a response.

Materials & experimental systems

n/a	Involved in the study
☒	☐ Antibodies
☒	☐ Eukaryotic cell lines
☒	☐ Palaeontology and archaeology
☒	☐ Animals and other organisms
☒	☐ Human research participants
☒	☐ Clinical data
☒	☐ Dual use research of concern

Methods

n/a	Involved in the study
☒	☐ ChIP-seq
☒	☐ Flow cytometry
☒	☐ MRI-based neuroimaging
